# Tryptanthrin Nanoliposomal Lotion as a Corticosteroid-Free Strategy for Safe and Effective Treatment of Atopic Dermatitis

**DOI:** 10.34133/bmr.0384

**Published:** 2026-07-24

**Authors:** Yo Han Song, Young-Guk Na, Moon Sup Yoon, Da-Eun Kim, Suyeon Hwang, Samuel Lee, Young Ju Jo, Ji Hyun Bang, Gabsik Yang, Dong-Sung Lee, Hong-Ki Lee, Cheong-Weon Cho

**Affiliations:** ^1^ College of Pharmacy, Chungnam National University, Daejeon 31434, Republic of Korea.; ^2^ College of Korean Medicine, Woosuk University, Jeonju, Republic of Korea.; ^3^ Research Institute of Pharmaceutical Sciences, College of Pharmacy, Chosun University, Gwangju, Republic of Korea.; ^4^ College of Veterinary Medicine, Chungbuk National University, Cheongju 28644, Republic of Korea.

## Abstract

Atopic dermatitis is a chronic inflammatory skin disease characterized by dry skin, itching, and recurrent eczematous lesions. Although current therapeutic strategies are effective, their use is often limited due to adverse effects. Therefore, the development of safer and more effective alternatives is required. Tryptanthrin is a yellow-gold alkaloid compound with anti-atopic and various pharmacological activities. However, its poor aqueous solubility results in low topical bioavailability. A tryptanthrin-loaded liposomal lotion was developed using a design of experiments approach to improve topical delivery. The optimized formulation showed enhanced physicochemical stability and moisturizing properties, as well as improved drug release compared to free tryptanthrin. In human keratinocyte cells, the liposomal lotion exhibited lower cytotoxicity than the free tryptanthrin and demonstrated higher skin residual. In the DNCB-induced atopic dermatitis mouse model, the tryptanthrin-loaded liposomal lotion produced therapeutic effects comparable to or greater than those of 1.0% hydrocortisone, without inducing adverse effects such as skin atrophy. Overall, the tryptanthrin-loaded liposomal lotion is presented as a promising and safe strategy for the topical treatment of atopic dermatitis.

## Introduction

Atopic dermatitis (AD) is a common inflammatory skin disorder characterized by dry, itchy skin and recurrent eczema lesions. The estimated global prevalence of AD is around 2.0% in adults and 4.0% in children, with rates of 2.8% for females and 2.4% for males [1]. Although the pathogenesis of AD remains incompletely understood, thymic stromal lymphopoietin (TSLP) has been identified as a pivotal contributor to its development [2]. TSLP positively correlates with AD and is highly expressed by keratinocytes in acute and chronic AD-associated skin lesions. Furthermore, TSLP concentrations are markedly elevated in both adult and pediatric AD patients compared to healthy individuals [3,4].

The activation of the inflammasome by an allergen causes caspase-1 activation within a cytosolic multi-adaptor complex in an inflammasome-specific manner, which subsequently drives the maturation and secretion of pro-inflammatory cytokines and regulates both innate and adaptive immune responses [2]. Caspase-1 activates the inactive precursor of interleukin-1β (IL-1β), which is essential for inflammation [5]. Receptor-interacting protein 2 (Rip2) regulates caspase-1, which is activated within the inflammasome [6]. Also, Rip2 activates the nuclear factor κ-light-chain-enhancer of activated B cells (NF-κB) pathway that mediates immune and inflammation responses. Caspase-1 can activate NF-κB through Rip2, and NF-κB binds to the promoter of the TSLP gene, regulating its transcription [7]. Consequently, TSLP expression leads to increased inflammatory cell infiltration, elevated CD4^+^ T helper 2 (Th2) cell counts, and higher immunoglobulin E (IgE) concentration, ultimately contributing to the development of AD [8].

AD treatment is categorized into topical and systemic approaches. For topical treatment, ointments containing corticosteroids like hydrocortisone (HC) or calcineurin inhibitors such as tacrolimus and pimecrolimus are used [9]. Subcutaneous injection with IL-4 and IL-13 inhibitors, such as dupilumab, and tablets with Janus kinase (JAK) inhibitors, including abrocitinib, are used for systemic treatment [10,11]. Topical steroids may cause adverse effects, including skin atrophy, discoloration, and purpura [12]. Topical calcineurin inhibitors effectively reduce inflammation and itching without causing skin atrophy [13]. However, their use can lead to localized burning sensations, and concerns have been raised about potential immunosuppression and an increased risk of cancer with long-term application [14,15]. In addition, biological agents, including dupilumab, have good therapeutic effects, but they are expensive and have side effects of conjunctivitis and dermatitis [16]. To overcome these limitations, the development of new therapeutic strategies that are both safer and more effective is required.

Tryptanthrin (TT) is a weakly basic, golden-yellow alkaloid compound with various pharmacological activities, including anti-inflammatory, antibacterial, anticancer, antiprotozoal, antioxidant, and antifungal effects [17]. Notably, it has been shown to treat AD by down-regulating IL-1β and TSLP expression through inhibition of the Rip2/caspase-1/NF-κB pathway [2]. Despite its numerous pharmacological values, the low aqueous solubility of TT (1.3 μg/ml) significantly limits its topical bioavailability [18]. Consequently, the development of TT formulation for topical application remains a significant challenge.

Hyaluronic acid (HA) is a glycosaminoglycan composed of repeated disaccharide units of N-acetylglucosamine and d-glucuronic acid. HA is a component of connective, epithelial, and neural tissues and constitutes a significant portion of the extracellular matrix [19]. Due to HA stability profile, moisturizing potential, and physicochemical properties, HA is widely used in various biomedical applications, including the cosmetic industry, drug delivery, and wound healing [19].

Nanocarrier-based drug delivery systems such as nanostructured lipid carriers, solid lipid nanoparticles, and liposomes present a promising strategy to enhance drug penetration through the skin while minimizing drug-related side effects [20]. They have been shown to enhance dermal drug penetration without skin barrier disruption, target the drug to the skin with no systemic absorption, prolong the drug release, and allow less frequent administration [21]. Liposomes are vesicles consisting of one or more phospholipid bilayers that enclose an aqueous compartment, providing a versatile platform for the encapsulation and delivery of various drugs. Due to their lipid composition, which closely resembles that of cell membranes, liposomes can effectively penetrate the stratum corneum barrier, facilitating deeper drug delivery into the epidermis and dermis layers of the skin [22,23]. One of the major challenges associated with liposomal formulations is their low colloidal stability. To address the stability issues inherent to conventional liposomes, the incorporation of hydrophilic polymers has emerged as an effective strategy. Hydrophilic polymers introduce steric barriers between adjacent bilayers, preventing aggregation and improving stability [24]. Polyethylene glycol (PEG) is one of the most widely utilized polymers for liposome stabilization, primarily enhancing in vivo stability by minimizing interactions between liposomes and plasma proteins during intravenous administration [25]. Additionally, PEG has been used in liposomes designed for topical applications, where it has been shown to enhance liposomal stability without affecting the drug’s penetration into the skin [26].

Microfluidic technology (MF) offers a promising platform for the fabrication of lipid-based nanoparticles, including liposomes [27]. This technique utilizes various channel geometries and incorporates microfluidic effects such as shear stress and electric fields within the mixing channels [28]. Previous studies have reported the successful formulation of TT-loaded ethosomes utilizing MF and phospholipids. In particular, MF enables precise control over the mixing of organic and aqueous phases, resulting in nanoparticles with consistent size, morphology, and structural uniformity [23].

In this study, TT-loaded liposomes (TLs) were prepared using MF. Additionally, a TL lotion (TLL) formulation with enhanced viscosity, incorporating HA, was developed and evaluated for its potential application in AD treatment. The liposomal components included soy-l-phosphatidylcholine (Soy-PC), cholesterol (Chol), 1,2-dimyristoyl-rac-glycero-3-methoxypolyethylene glycol-2000 (DMG-PEG), and TT. In designing the formulation, each excipient was selected according to a defined functional role. Soy-PC was used as the principal bilayer-forming phospholipid to provide a biocompatible matrix for the encapsulation of poorly water-soluble TT and for topical vesicle formation [22,23]. Chol was incorporated to increase lipid packing and membrane rigidity and to reduce bilayer permeability, thereby improving vesicle stability [29]. DMG-PEG was included to provide steric stabilization and reduce vesicle aggregation during storage [24–26]. For topical application, HA and Carbopol 971 NF were selected to provide hydration/barrier-supportive effects and a semisolid viscous vehicle suitable for local skin residence [19,30]. Accordingly, the objective of this study was not to introduce a fundamentally new biomaterial class, but to engineer a TT-loaded PEGylated liposomal topical system with optimized colloidal properties and to translate it into a clinically applicable lotion format for local AD treatment. The TL formulation was optimized using Box–Behnken design (BBD), which is a statistical method within the design of experiments approach.

## Materials and Methods

### Materials

TT was purchased from BLD Pharmatech Ltd. (Songjiang District, Shanghai, China). DMG-PEG and Soy-PC were purchased from Avanti Polar Lipids (Alabaster, AL, USA). Acetone, Chol, dimethyl sulfoxide (DMSO), ethanol (EtOH), high-performance liquid chromatography (HPLC)-grade acetonitrile (ACN), olive oil, and polysorbate 80 (Tween 80) were purchased from Samchun Chemical (Pyungtaek, Republic of Korea). Carbopol 971 NF polymer was purchased from Lubrizol Advanced Materials Inc. (Cleveland, OH, USA). Sodium hyaluronate (HA) was presented by Bioland Ltd. 1-Chloro-2,4-dinitrobenzene (DNCB), 3-(4,5-dimethylthiazol-2-yl)-2,5-diphenyltetrazolium bromide (MTT), and 4-(1,1,3,3-tetramethylbutyl) phenyl-polyethylene glycol (Triton X-100) were purchased from Sigma-Aldrich (St. Louis, MO, USA). Phosphate-buffered saline (PBS) was purchased from LPS solution (Daejeon, Republic of Korea). HaCaT cells were obtained from the Korean Cell Line Bank (Seoul, Korea). According to the supplier, authentication and mycoplasma testing were performed prior to distribution. Dulbecco’s modified Eagle’s medium (DMEM), fetal bovine serum (FBS), penicillin–streptomycin, and trypsin–EDTA were purchased from Gibco BRL (Gaithersburg, MD, USA). Formaldehyde solution (formalin) was purchased from Junsei Chemical Co. Ltd. (Tokyo, Japan). Distilled water (DW) was used throughout the experiments.

### Preparation of TL and TLL

#### Preparation of TL

TL was prepared using a reopenable and washable microfluidic chip, which comprises 2 inlets and one outlet (Neo Nanotech Co. Ltd., Daejeon, Republic of Korea). The microfluidic chip consists of an upper plate and a lower plate, with the upper plate made of polycarbonate and the lower plate made of thermoplastic polyurethane. Briefly, TL was prepared by injecting an EtOH solution containing Soy-PC, Chol, DMG-PEG, and TT into the right inlet channel at a flow rate of 1 ml/min while simultaneously injecting PBS (pH 7.4) into the left inlet channel at a flow rate of 3 ml/min. The resulting formulation was collected from the outlet channel. Blank liposome (BL) was prepared using the same method as TL, without TT.

#### Preparation of TLL

TLL was prepared using 1.0% (w/v) Carbopol 971 NF and 0.5% (w/v) HA into TL. BL lotion (BLL) was prepared using the same method as TLL, with BL used in place of TL. Initially, a homogeneous solution consisting of PBS, BL, or TL was prepared in specific proportions. Carbopol 971 NF and HA powders were then evenly dispersed into the solution and allowed to swell gradually and uniformly overnight. The following day, hydrochloric acid (HCl) and sodium hydroxide (NaOH) were added to neutralize Carbopol 971 NF and HA and achieve a pH of approximately 5.5 to facilitate stable lotion formation. Simultaneously, the mixture was vigorously stirred to ensure even distribution of HCl and NaOH, yielding the BLL and TLL.

### The optimization of TL

BBD is suitable for optimizing parameter levels through analysis of response surface design. The experimental design and statistical analysis of BBD were conducted using the Design Expert 13 software (Sta-Ease Inc., Minneapolis, MN, USA). Experiments with BBD were designed to have 3 factors and 3 responses. The amount of Soy-PC (*X*_1_, μmol), the ratio of Chol to Soy-PC (*X*_2_, %), and the ratio of DMG-PEG to Soy-PC (*X*_3_, %) were set within the ranges indicated in Table 1. The responses based on the factors were set as droplet size within the range (*Y*_1_, nm), minimum polydispersity index (PDI; *Y*_2_), and maximum encapsulation efficiency (EE; *Y*_3_, %). After setting up the range of each factor, randomized 17 runs were suggested, and optimized responses were analyzed with a quadratic model. The model presented for each response was analyzed by analysis of variance (ANOVA). Statistical parameters such as *P* value, lack of fit, *R*^2^, adjusted *R*^2^, and adequate precision were evaluated to optimize the fitting model. The most optimal composition was selected as the optimized formulation and the statistically optimized TL formulation. Next, a reproducibility test was performed to compare the error rate between actual value and predicted value.

**Table 1.** Factors and responses used in Box–Behnken design

Factors	Range	
	Low limit	High limit
*X*_1_: Soy-PC amount (μmol)	10	40
*X*_2_: Chol/Soy-PC ratio (%)	0	50
*X*_3_: DMG-PEG/Soy-PC ratio (%)	2.5	20
Responses	Constraints	
	Goal	Importance
*Y*_1_: Droplet size (nm)	In range	+
*Y*_2_: Polydispersity index	Minimize	+
*Y*_3_: Encapsulation efficiency (%)	Maximize	+++++

### Characterization of TL and TLL

#### Droplet size and PDI

Droplet size and PDI of liposomes were evaluated using an electrophoretic light scattering analyzer (ELSZ-2000; Otsuka Electronics Co., Osaka, Japan). For measurement, the samples were diluted to ^1^/_10_ concentration with deionized water. Droplet size and PDI were measured under light scattering conditions. Droplet size and PDI were measured 3 times with 30 runs each.

#### EE determination

EE (%) was measured by completely breaking the lipid membrane of the final liposomes. Briefly, 100 μl of the prepared liposomes was mixed with Triton-X and DW. After that, ACN was added, and cold bath sonication for 10 min was applied to allow sufficient drug to come out. Centrifugation was performed at 848*g* for 20 min at 4 °C. The supernatant was collected and analyzed according to the HPLC analysis conditions. EE (%) was calculated as follows:

$$\begin{array}{c} \mathrm{EE}\left(\%\right)=\left(\text{Amount of}\;\mathrm{TT}\;\text{loaded into liposome}\right)/\\ \left(\text{Total amount of}\;\mathrm{TT}\right)\times 100 \end{array}$$ (1)

#### HPLC analysis condition of TT

TT was analyzed by HPLC using an Agilent 1260 Infinity II Series (Agilent Technologies Inc., Santa Clara, CA, USA) consisting of ultraviolet–visible detector, pumps, thermostats, and autosampler. The column was equipped with YMC-Pack CN (250 × 4.6 mm, 5 μm, YMC Co. Ltd., Kyoto, Japan), and the mobile phase was DW and ACN (55:45, v/v). The injection volume and the flow rate were 10 μl and 0.8 ml/min, respectively. The temperature of the column was maintained at 35 °C. The detection wavelength was 254 nm.

#### Transmission electron microscopy

The morphology of TL was examined using transmission electron microscopy (TEM) (Tecnai G2 F30 S-Twin, FEI, Hillsboro, OR, USA). TL was diluted 10-fold with DW. The diluted TL (10 μl) was loaded onto a carbon-coated copper grid for 1 min, followed by negative staining using UranyLess solution (Electron Microscopy Sciences, Hatfield, PA, USA) for another 1 min. The negatively stained grid was dried overnight at room temperature (RT) and observed under the microscope at 300 kV.

#### Cryogenic transmission electron microscopy

The morphology of TL and TLL was examined using cryo-TEM (Glacios Cryo-TEM, Thermo Fisher Scientific, Waltham, MA, USA). TL and TLL were diluted 10-fold with DW. TL and TLL were vitrified using a Vitrobot system (Thermo Fisher Scientific, Waltham, MA, USA). Briefly, 10 μl of the diluted TL and TLL was applied onto a glow-discharged carbon-coated copper grid, followed by controlled blotting to remove excess liquid and form a thin aqueous film. The grid was then rapidly plunge-frozen into liquid ethane cooled by liquid nitrogen to achieve vitrification, preserving the native hydrated structure of the nanoparticles. The vitrified grids were prepared at 4 °C and 100% humidity and subsequently imaged under a cryo-electron microscope operated at 200 kV.

#### Differential scanning calorimetry

The heat flow was measured using differential scanning calorimetry (DSC) (N-650 model, Scinco, Seoul, Republic of Korea) to confirm the thermal transition state. DSC analysis was performed on Soy-PC, Chol, DMG-PEG, the physical mixture, BL, and TL. The sample (2 mg) was placed in an aluminum DSC pan and analyzed. The temperature range was 30 to 300 °C, and the heating temperature was 10 °C/min under nitrogen gas.

#### Fourier transform infrared spectroscopy

The Fourier transform infrared (FT-IR) spectra were conducted using ALPHA-P FT-IR spectrometer (Bruker Optics Inc., Billerica, MA, USA) to determine the chemical interaction between Soy-PC, Chol, DMG-PEG, physical mixture, BL, and TL. The spectra were obtained from 4,000 cm^−1^ to 600 cm^−1^.

#### Stability study

The stability of TL was determined at a storage of 4 °C and RT for up to 13 d. The physical and chemical stability of TL was evaluated. Physical stability was determined through visual observation, droplet size (nm), and PDI determination. Chemical stability was determined through EE (%). For TL stored at 4 °C, additional chemical stability evaluations were conducted on days 0, 7, and 13 through DSC analysis.

The stability of TLL was determined at a storage of 4 °C for 4 weeks. At regular time points (0, 7, 14, 21, and 28 d), samples of TLL were collected to monitor the changes in lotion appearance, pH, droplet size (nm), PDI, and EE (%). TLL solution was appropriately diluted for analysis. The pH of TLL was determined using an Orion Star A211 pH meter (Thermo Fisher Scientific, Waltham, MA, USA).

### Moisture loss study

Moisture loss was measured by modifying a previously reported gravimetric method [31]. Briefly, an agar medium was prepared by pouring 0.8% (w/v) agar solution into 90 × 15 mm Petri dishes and allowing it to solidify. For comparison, the TT solution prepared in PBS was used as a control, and TL and TTL formulations were applied to the surface of the agar medium. The Petri dishes were stored at RT and 37 °C. The weight of each sample was measured at predetermined time points (0, 1, 2, 4, 6, 8, 12, 24, and 48 h), and the moisture loss was calculated accordingly. Moisture loss was calculated by the following equation:

$$\text{Moisture loss}\left(\%\right)=\left(W_{0}-W_{t}\right)/W_{0}\times 100$$ (2)

In this equation, *W*_0_ is the initial weight of the sample at time zero, and *W_t_* is the weight of the same sample at a specific time point *t*. All experiments were performed in 3 replicates (*n* = 3). Evaporation constant (*K*) and the time required for 50% moisture loss (*H*_½_) were calculated by the nonlinear regression of Prism 7(GraphPad Software, La Jolla, CA, USA).

### In vitro release study

To perform the in vitro release profile of TL and TLL, a Franz diffusion cell (Labfine, Gunpo, Korea) with a diffusion area of 1.77 cm^2^ and a receptor volume of 12.5 ml was used. Molecular cutoff dialysis membranes (10 kDa) (Viskase Inc., Lombard, IL, USA) were used for the in vitro release studies, which were stabilized in DW for 12 h before use. The donor compartment was secured using a clamp. The receptor compartment was filled with buffer solutions at pH 5.5, 7.4, or 8.5 containing 40.0% (v/v) EtOH and 1.0% (v/v) Tween 80, and maintained at 37 °C with stirring at 500 rpm. Air bubbles in the receptor compartment were carefully removed before starting the experiment. After a 2-h equilibration period, free TT, TL, and TLL, each corresponding to 0.325 mg of TT, were applied to the surface of the donor compartment. Samples (1 ml) were withdrawn from the receptor compartment at specific time intervals (0, 1, 2, 4, 6, 8, 12, 24, 36, and 48 h) and filtered through a 0.45-μm polyvinylidene difluoride (PVDF) syringe filter (Hyundai Micro, Seoul, Republic of Korea). After each sampling, an equal volume of fresh buffer solution was added to maintain the receptor volume. Drug concentrations in the collected samples were quantified using HPLC analysis.

### Skin permeation and residual studies

Skin permeation and retention of free TT, TL, and TLL were performed using Franz diffusion cells as previously described in detail (see the Moisture loss study section). Forty male Sprague–Dawley rats (7 weeks, weighing 200 to 250 g) were maintained under standard laboratory conditions (22 °C, 40% relative humidity, 12 h light/dark cycle) with free access to food and water. All animal experiments were reviewed and approved by the Local Ethical Committee of Chungnam National University (approval no. 202503A-CNU-036) and conducted in accordance with the guidelines of the Chungnam National University Institutional Animal Care and Use Committee (Daejeon, Republic of Korea). The rats were euthanized using medical-grade CO₂ in a dedicated chamber (gradual-fill displacement), and the shaved skin was excised. Before the experiment, subcutaneous fat was carefully removed, and the skin was cut into 2 × 2 cm^2^. The stratum corneum side was exposed to the donor compartment, which was then clamped. The receptor compartment was filled with pH 7.4 buffer solution containing 1.0% (v/v) Tween 80 and stirred at 37 °C to maintain a skin temperature of 32 °C. Air bubbles in the receptor compartment were carefully removed before starting the experiment. After a 2-h equilibration period, TT, TL, and TLL, each corresponding to 0.5 mg of TT, were applied to the surface of the donor compartment. Samples (1 ml) were withdrawn from the receptor compartment at specific intervals (0, 1, 2, 4, 6, 8, 12, 24, 36, and 48 h) and filtered through a 0.45-μm PVDF syringe filter. An equal volume of fresh medium was added to the receptor compartment after each sampling to maintain the volume. The TT content in the samples was analyzed using HPLC, and the cumulative amount over time was plotted. The permeability of the drug was calculated using the following formula:

$$\begin{array}{c} \text{Skin permeation of drug}\left(\%\right)=\\ \left(\text{Amount of permeated drug}/\text{Initial amount of drug}\right)\times 100 \end{array}$$ (3)

At specific intervals (12, 24, 36, and 48 h), the residual drug remaining in the donor compartment was collected to quantify the amount of TT that had not permeated. Then, the skin was carefully removed, and the upper skin layer was washed with a swab moistened with ACN to measure the residual amount of TT in the stratum corneum. Subsequently, the skin was placed in DW at 60 °C for 2 min to separate the dermis and epidermis. The epidermis and dermis layers were carefully separated using surgical tweezers and cut into small pieces. Each layer was homogenized in 2 ml of a DMSO and ACN (1:1) mixture for 5 min. The homogenate was shaken overnight in a water bath to extract TT from each layer. The mixture was centrifuged at 16,000*g* for 5 min, and the supernatant was diluted with ACN. The solution was filtered through the 0.45-μm PVDF syringe filter and analyzed using HPLC.

### In vivo study

#### Animals and AD model

Male BALB/c mice (7 weeks of age, weighing 20 to 25 g) were purchased from Orient Bio Inc. (Seongnam, Republic of Korea). All animals were maintained under standard laboratory conditions (22 °C, 40% relative humidity, 12 h light/dark cycle) with free access to food and water. All animal experiments were reviewed and approved by the Local Ethical Committee of Chungnam National University (approval no. 202503A-CNU-036) and conducted in accordance with the guidelines of the Chungnam National University Institutional Animal Care and Use Committee (Daejeon, Republic of Korea).

DNCB-induced AD is a standard model as it displays many disease-associated characteristics of human AD. BALB/c mice were randomly divided into 5 groups (*n* = 4 per group, *n* = 3 for control group) composed of control (C), negative control (NC), 1.0% HC, BLL, and TLL groups. To induce AD in the NC, HC, BLL, and TLL groups, DNCB was applied. A solution of DNCB mixed with a vehicle consisting of acetone and olive oil (3:1) was used to establish the pathological model. BALB/c mice were acclimated to laboratory conditions for 1 week. After carefully shaving the dorsal skin of the mice, the area was left undisturbed for 24 h to permit healing of micro-wounds. For 2 weeks, 1.0% DNCB solution was applied to the dorsal and ear regions twice a week. Subsequently, 0.5% DNCB solution was applied to the same areas twice a week for an additional 4 weeks to induce AD. In the control group, the vehicle was applied to the dorsal and ear regions in the same volume and schedule. To evaluate the therapeutic efficacy of TLL for AD, drugs were applied daily during the 4 weeks of 0.5% DNCB application (Fig. S1). PBS, 1.0% HC, BLL, and TLL were applied to the dorsal and ear regions of mice in the NC, HC, BLL, and TLL groups, respectively. The 1.0% HC cream, a mild topical steroid, was used as a clinically relevant comparator to contextualize the therapeutic efficacy and safety of TLL, rather than to establish dose equivalence with TT. Following terminal procedures, mice were humanely euthanized using medical-grade CO₂ in a regulated, gradual-fill euthanasia chamber in accordance with the approved institutional animal care and use committee protocol.

#### Ear thickness and AD scoring

Ear thickness was measured at 1-week intervals on the right ear of mice using a vernier caliper. The scoring of AD was performed using a visual assessment method. The skin scores based on sensory evaluation were assigned by at least 2 independent observers, and the average score was calculated. The evaluation parameters included erythema/hemorrhage, scarring/dryness, edema, and excoriation. Each parameter was graded as none (0), mild (1), moderate (2), or severe (3), resulting in a total score ranging from 0 to 12.

#### Measurements of IgE concentration, spleen length, body, and spleen weight

On the day of sacrifice, all mice (*n* = 20) were anesthetized with isoflurane (2% to 3% in O₂), and blood was collected from the retro-orbital plexus during euthanasia. Approximately 1.5 ml of blood was obtained from each mouse and transferred into EDTA-containing tubes. This was a single terminal blood draw at study end (no repeated intervals). The sample was then centrifuged at 5,000 rpm for 5 min at RT to separate the plasma. The plasma was stored at −80 °C until use and thawed just before the experiment. IgE was measured using the mouse IgE enzyme-linked immunosorbent assay (ELISA) kit (Invitrogen, Carlsbad, CA). All samples were appropriately diluted with Assay Diluent of IgE ELISA kit prior to measurement. The spleen length was measured using ImageJ software. The body weight and spleen weight of the mice were measured on the day of sacrifice and expressed as the spleen index. The spleen index was calculated using the following formula:

$$\text{Spleen index}\left(\mathrm{mg}/g\right)=\text{Spleen weight}\left(\mathrm{mg}\right)/\text{Body weight}\left(g\right)$$ (4)

#### Measurements of IL-1β and TSLP concentration

IL-1β and TSLP were measured using the mouse IL-1β and mouse TSLP ELISA kit (Invitrogen, Carlsbad, CA, USA). All samples were appropriately diluted with Assay Diluent of ELISA kit prior to measurement.

#### H&E and toluidine blue stain

Hematoxylin and eosin (H&E) and toluidine blue staining were performed to evaluate histological changes. On the day of sacrifice, AD-induced mice from each group were humanely euthanized, and dorsal skin tissues from the AD-induced area were excised into 2 × 2 cm^2^ sections. The excised dorsal skin tissues were then fixed in neutral buffered formalin and subsequently trimmed into appropriate sizes (2 to 3 mm thick) for histological processing. The trimmed tissue samples were placed in labeled cassettes and processed using a tissue processor for 13 h. The processed tissues were then embedded in paraffin, sectioned into 3- to 4-μm-thick slices using a microtome, and mounted on slides. The slides were dried, deparaffinized, rehydrated, and washed with DW. Finally, the sections were stained with either H&E or toluidine blue.

Epidermal and dermal thicknesses and immune cell infiltration were assessed by H&E staining. Toluidine blue staining was used to evaluate mast cell infiltration in skin tissues. For histological evaluation, skin samples were collected from each group (*n* = 3), and the average value was calculated from 3 different sites per mouse.

#### NF-κB immunohistochemical staining

Immunohistochemical staining for NF-κB p65 was additionally performed on paraffin-embedded dorsal skin sections to evaluate inflammation-associated NF-κB activity in lesional skin. After deparaffinization, rehydration, antigen retrieval, and blocking, sections were incubated with an anti-NF-κB p65 primary antibody (Santa Cruz Biotechnology, sc-8008; RELA/NF-κB p65), followed by an appropriate secondary antibody and 3,3′-diaminobenzidine (DAB)-based chromogenic detection with hematoxylin counterstaining. Digital image analysis was performed using QuPath software to quantify DAB-positive area (%) and DAB-positive cell nuclei (%) in representative lesional fields from each group. These parameters were used to evaluate overall NF-κB p65 immunoreactivity and nuclear localization in skin tissue.

### Statistical analysis

All results are provided as mean ± standard deviation (SD). Statistically significant differences between groups were determined by ANOVA with Prism 7. The mean was compared by Student’s *t* test or 2-way ANOVA. *P < 0.05, **P < 0.01, ***P < 0.001, and ****P < 0.0001 were statistically significant.

## Results and Discussion

### The optimization of TL

The optimized TL was formulated using the BBD statistical analysis that was conducted to determine the relationship between the proposed model and the response through the Design Expert 13 software. In liposomal formulation design for drug delivery to skin, the composition of the liposomes is the most crucial element, because the interactions of liposomes with the skin and the transfer of drug from the liposomes to the skin depend on the thermodynamic state of the liposomal bilayers, and hence on the nature of the phospholipids used in their preparation [22]. These formulation variables were selected because they represent key structural determinants of liposomal performance: Soy-PC for bilayer formation, Chol for membrane stabilization, and DMG-PEG for steric stabilization. Therefore, in this study, the amounts of Soy-PC (*X*_1_, μmol), the ratio of Chol to Soy-PC (*X*_2_, %), and the ratio of DMG-PEG to Soy-PC (*X*_3_, %) were selected as the 3 factors. Additionally, the responses were set as droplet size (*Y*_1_, nm), PDI (*Y*_2_), and EE (*Y*_3_, %). Droplet size and size distribution are important evaluation factors for nanoparticles because droplet size and size distribution influence drug release rate and biodistribution. Also, small particles under 200 nm exhibit higher skin retention [32,33]. A high EE value allows for the delivery of a larger amount of the drug. Therefore, *Y*_1_ was set within the range of 200 nm or less, *Y*_2_ was set to minimize, and *Y*_3_ was set to maximize.

**Fig. 1.**
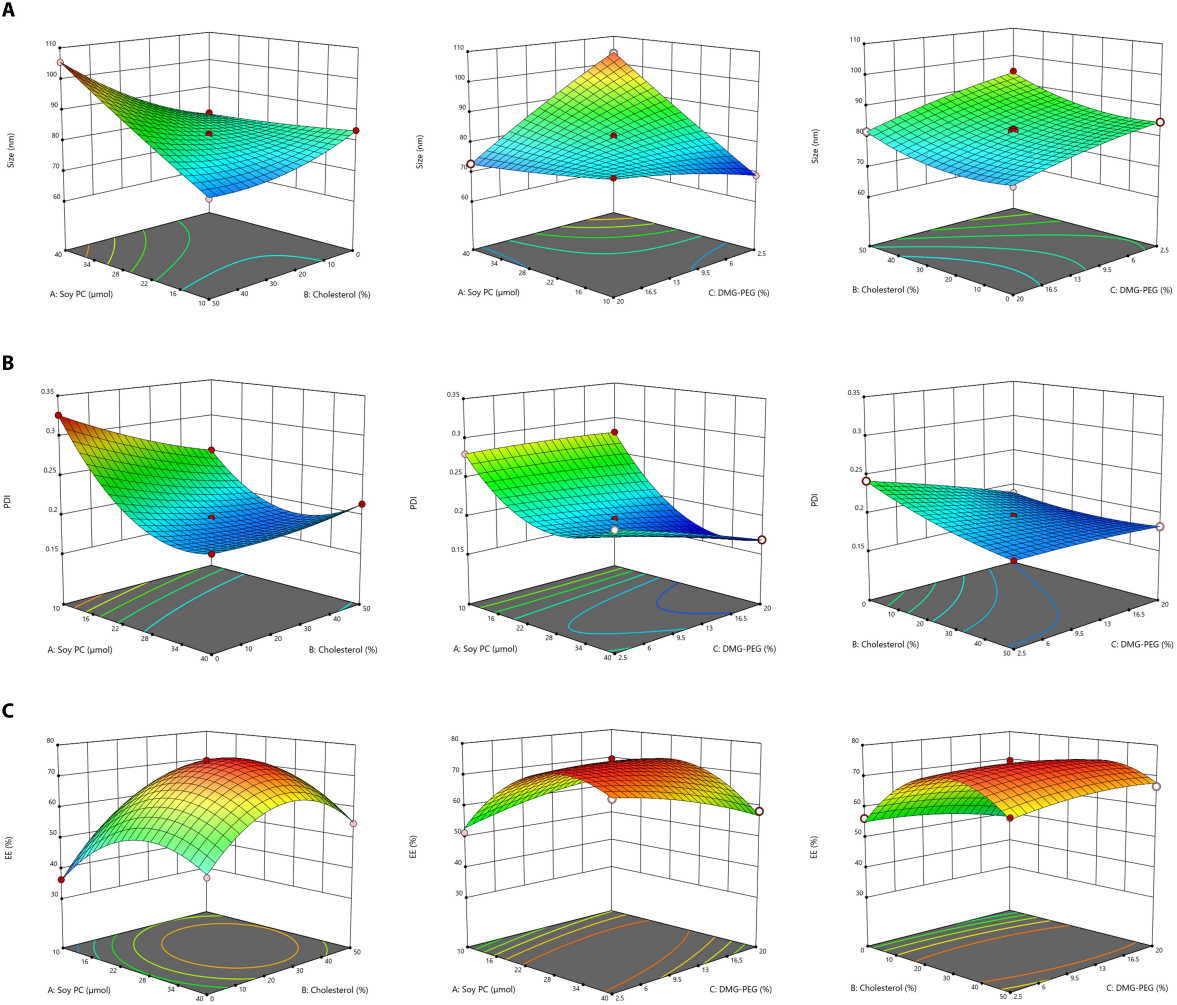
3D surface plots of responses: (A) droplet size, (B) PDI, and (C) EE.

Table S1 shows the 17 experiments and data obtained using the BBD design. The distributions of droplet size, PDI, and EE values were 68.8 to 105.4 nm, 0.169 to 0.326, and 36.28 to 75.64%, respectively. Statistical parameters were investigated to determine the appropriate model, and each parameter was presented in Table S2. All responses (*Y*_1_, *Y*_2_, and *Y*_3_) were fitted to quadratic models, and ANOVA results indicated that the models were significant. The model *P* value was <0.0001, which indicated that the model was significant. Lack of fit *P* values exceeded 0.05, making it suitable for explaining the model’s reliability and association in *Y*_1_, *Y*_2_, and *Y*_3_ [34,35]. The *R*^2^, adjusted *R*^2^, and predicted *R*^2^ values indicate the degree to which the response of the proposed model matches the experimental data. If the adjusted *R*^2^ value is high and close to the *R*^2^ value, the model is suitable for predicting the response [36]. Additionally, the model is suitable when the difference between the adjusted *R*^2^ and predicted *R*^2^ is less than 0.2 [37]. For all responses, the adjusted *R*^2^ value was higher than 0.9 and similar to the *R*^2^ value. The difference between the adjusted *R*^2^ and predicted *R*^2^ was less than 0.2, indicating that the experimental results were statistically significant. Adequate precision measures the signal-to-noise ratio, and a value greater than 4 is desirable [38]. For Y₁, Y₂, and Y₃, the adequate precision values were 82.9617, 416.0444, and 36.6717, respectively, all of which were higher than 4. The relationship of 3 factors and 3 responses was described by 3-dimensional (3D) surface plots and coefficient equations (Fig. 1 and Table S3). Synergistic effects are indicated by positive coefficients, and antagonistic effects are indicated by negative coefficients [39]. Since droplet size, PDI, and EE values follow a quadratic model, they were influenced by all factors. Droplet size was most influenced by the amount of Soy-PC. Droplet size decreased when the Soy-PC amount and Chol/Soy-PC ratio decreased, and the DMG-PEG/Soy-PC ratio increased. PDI was most influenced by the Soy-PC amount. PDI decreased when the Soy-PC amount, Chol/Soy-PC ratio, and DMG-PEG/Soy-PC ratio decreased. However, an increasing trend was also observed depending on the relative proportions of the factors. EE was most influenced by the Chol/Soy-PC ratio. EE increased when the Soy-PC amount and Chol/Soy-PC ratio increased and the DMG-PEG/Soy-PC ratio decreased. However, as mentioned earlier, since all responses are the quadratic model, it is difficult to describe the correlations in simple terms. The predicted values and actual values of the optimized TL are shown in Table 2. The optimized factors were set with 26.9 μmol of Soy-PC amount (*X*_1_), 31.6% of Chol/Soy-PC ratio (*X*_2_), and 11.4% of DMG-PEG/Soy-PC ratio (*X*_3_). The actual value was confirmed through the reproducibility test, and it showed with 80.8 ± 1.3 nm of droplet size, 0.178 ± 0.002 of PDI, and 72.1 ± 2.6% of EE values. When the actual values and the predicted values were compared, the error percent (%) was less than 10%. Thus, it indicated that TL was successfully optimized.

**Table 2.** Predicted and actual values of responses for the optimized TL (mean ± SD; *n* = 3)

Optimized factors	Soy-PC amount (μmol)	Chol/Soy-PC ratio (%)	DMG-PEG/Soy-PC ratio (%)
	26.9	31.6	11.4
Responses	Droplet size (nm)	PDI	EE (%)
95% Cl low predicted value	83.4	0.187	73.6
Predicted value	84.0	0.188	75.0
95% Cl high predicted value	84.6	0.189	76.5
Actual value	80.8 ± 1.3	0.178 ± 0.002	72.1 ± 2.6
Error percentage (%)	3.8	5.3	3.8

### Characterization of TL and TLL

#### Characterization of TL

**Fig. 2.**
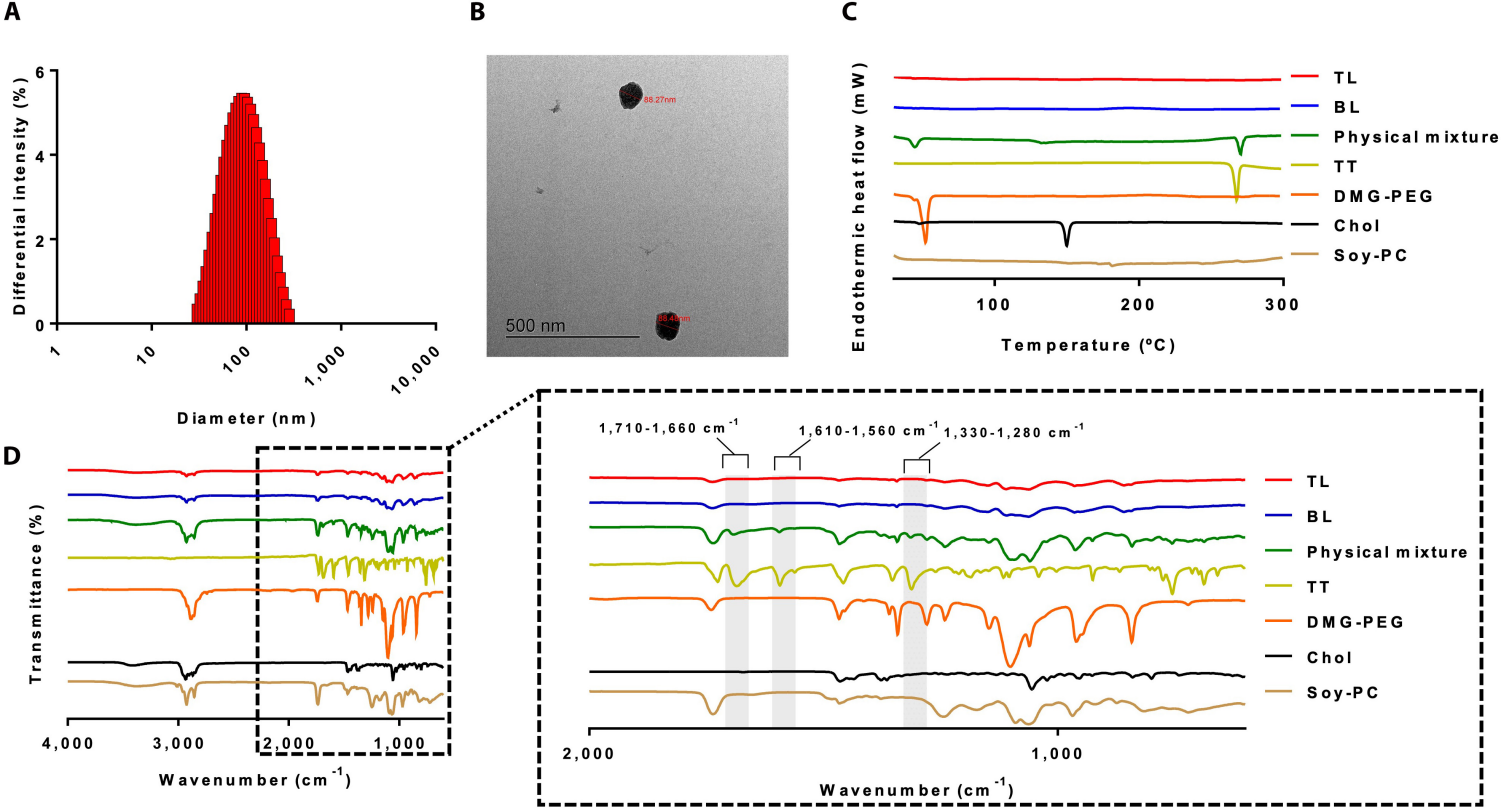
Physicochemical characterization and stability of TL. (A) Droplet size distribution and TEM image of the optimized TL. (B) TEM image of optimized TL. (C) DSC thermogram of Soy-PC, Chol, DMG-PEG, TT, physical mixture, BL, and TL. (D) FT-IR spectra of Soy-PC, Chol, DMG-PEG, TT, physical mixture, BL, and TL. The boxed region presents a magnified view of the 2,000 to 600 cm^−1^ range.

The physicochemical properties of TL were evaluated (Fig. 2). As mentioned in Table 2, the droplet size was 80.8 ± 1.3 nm, PDI was 0.178 ± 0.002, and EE was 72.1 ± 2.6%.

**Fig. 3.**
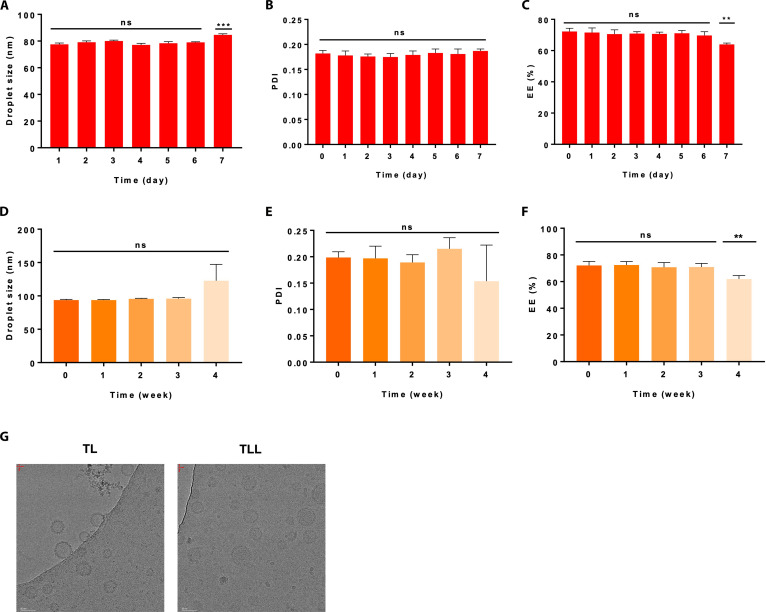
Stability of TL and TLL at 4 °C. (A) Droplet size of TL. (B) Polydispersity index (PDI) of TL. (C) Encapsulation efficiency (EE) of TL. (D) Droplet size of TLL. (E) PDI of TLL. (F) EE of TLL during storage at 4 °C. (G) Representative cryo-TEM images of TL and TLL in a near-native hydrated state, showing vesicle-like nanostructures and supporting the retention of liposomal morphology after incorporation into the lotion matrix. Values are expressed as mean ± SD (*n* = 3). **P < 0.01, ***P < 0.001.

The TEM images were conducted to confirm the morphology of TL and the actual size of TL (Fig. 2B). Through observation of TEM images of TL, it was confirmed that spherical TL with uniform size was prepared. Additionally, the droplet sizes observed in the TEM images were comparable to the measurements obtained from the ELSZ size analyzer. To further evaluate whether vesicular morphology was maintained after incorporation into the semisolid lotion base, cryo-TEM was additionally performed using TL and TLL (Fig. 3G). Unlike the negatively stained TEM image of TL, the cryo-TEM images provided morphological information in a near-native hydrated state. Vesicle-like spherical nanostructures were also observed in TLL, indicating that incorporation into the Carbopol 971 NF/HA-based lotion matrix did not cause obvious collapse of vesicular morphology.

To complement the limited field of view of the original negatively stained TEM image, comparative cryo-TEM images of TL and TLL were additionally obtained (Fig. 3G). These images provided a broader qualitative view of the hydrated formulations and enabled direct morphological comparison between the initial liposomes and the final lotion system. The comparative cryo-TEM images supported the presence of vesicle-like nanostructures in both TL and TLL.

DSC results were analyzed to confirm the crystallinity of TL through thermodynamic changes (Fig. 2C). TT exhibited an endothermic peak at 268.2 °C, while Soy-PC, Chol, and DMG-PEG displayed endothermic peaks at 181.6, 149.9, and 52.3 °C, respectively. In the physical mixture, both drug and excipient peaks were observed. The characteristic thermal analysis peaks of TT in TL were not observed. This suggests that the crystalline state of TT became completely amorphous in TL.

During the preparation of TL, the interaction between TT and other excipients was confirmed through FT-IR analysis. The FT-IR spectra for TT, Soy-PC, Chol, DMG-PEG, physical mixture, BL, and TL were presented in Fig. 2D. The FT-IR spectra of TT, the physical mixture, BL, and TL exhibit notable differences, suggesting successful drug encapsulation. The characteristic TT peaks appeared at 1,660 to 1,710 cm^−1^ (C=O carbonyl), 1,560 to 1,610 cm^−1^ (C=C aromatic), and 1,250 to 1,330 cm^−1^ (C–N amine). Comparing the FT-IR spectra between TT and TL revealed that the characteristic TT peak was absent in TL. This absence suggests that TT was effectively encapsulated within the phospholipid bilayer, rendering its individual spectral peaks undetectable. Characteristic TT peaks were observed in the physical mixture. Furthermore, the FT-IR profile of TL closely resembles that of BL, indicating that the characteristics of TT were masked within the liposome formulation.

#### Stability of TL

The stability of TL was evaluated based on changes in droplet size, PDI, and EE (Fig. 3A to C). TL and TL without DMG-PEG (TL^-PEG^) were stored at RT and 4 °C for up to 13 d. At predetermined time points, each stored sample was dispersed in DW and buffer solution for analysis. Immediately after preparation, the droplet size, PDI, and EE of TL were 78.3 ± 0.8 nm, 0.182 ± 0.006, and 72.2 ± 2.1%, respectively. For TL^-PEG^, these values were 84.6 ± 0.9 nm, 0.229 ± 0.015, and 44.7 ± 0.8%, respectively. When stored at RT, the droplet size, PDI, and EE of TL remained stable until days 2, 4, and 2, respectively, with aggregation occurring on day 6 (Fig. S2A to C). When stored at 4 °C, TL maintained stability until days 6, 7, and 6, respectively, with aggregation occurring on day 13. For TL^-PEG^ stored at RT, PDI remained stable until day 2. However, droplet size and EE failed to maintain their initial values from the first day, with aggregation occurring on day 4 (Fig. S2D to F). When stored at 4 °C, droplet size and PDI remained stable until day 1, whereas EE showed no stability from the initial state, with aggregation observed on day 7 (Fig. S2G to I). Overall, TL exhibited stability for 2 and 6 d at RT and 4 °C, respectively. In contrast, TL^-PEG^ failed to maintain its initial state at any storage conditions, showing no stability beyond 1 d.

DSC analysis was further conducted to evaluate the thermal property changes of TL stored at 4 °C (Fig. S3). Immediately after preparation, the amorphous nature of TL was confirmed, consistent with previous observations. On day 7, when droplet size and EE showed changes compared to their initial values, a slight endothermic shift was observed around 43.7 °C, resembling the thermodynamic properties of DMG-PEG. On day 13, when aggregation occurred, a more pronounced endothermic change was observed at approximately 45.8 °C.

Through the stability study, it was confirmed that the droplet size of the liposomes gradually increased immediately after TL was manufactured. Additionally, TL demonstrated greater stability than TL^-PEG^, which can be attributed to the stabilizing effect of PEGylated lipids. Furthermore, DSC analysis indicated that the crystallinity of TL influenced its stability.

#### Stability of TLL

The stability of TLL was evaluated based on changes in droplet size, PDI, droplet size distribution, appearance, EE, and pH (Fig. 3D to F and Fig. S4). TLL was stored at 4 °C for 4 weeks immediately after preparation. At predetermined time points, each stored sample was dispersed in DW and buffer solution for analysis. Immediately after preparation, the droplet size, PDI, EE, and pH of TLL were 93.7 ± 1.2 nm, 0.199 ± 0.011, 72.1 ± 3.0%, and 5.5 ± 0.3, respectively. At each week, droplet sizes of TLL were 93.7 ± 0.9, 95.6 ± 0.8, 95.9 ± 1.6, and 122.8 ± 24.5 nm, respectively. At each week, PDI values were 0.199 ± 0.011, 0.197 ± 0.023, 0.189 ± 0.015, and 0.215 ± 0.021. At each week, EE values were 72.5 ± 2.9%, 70.8 ± 3.5%, 71.0 ± 2.7%, and 62.0 ± 2.6%, respectively. At each week, pH values were 5.6 ± 0.2, 5.4 ± 0.3, 5.4 ± 0.2, and 5.3 ± 0.2. The appearance of TLL remained unchanged for 4 weeks, with no precipitation or aggregation observed.

While appearance and pH remained stable throughout the storage period, droplet size, PDI, and EE remained stable only for 3 weeks. Although PDI decreased in the fourth week, droplet size distribution analysis confirmed instability. The droplet size and PDI of TLL were larger than those of conventional TL due to the presence of HA and Carbopol 971 NF. However, it exhibited 3.5-fold greater stability. This increase in stability may be attributed to the improved dispersion stability resulting from the viscosity enhancement by HA and Carbopol 971 NF.

### Moisture loss study

**Fig. 4.**
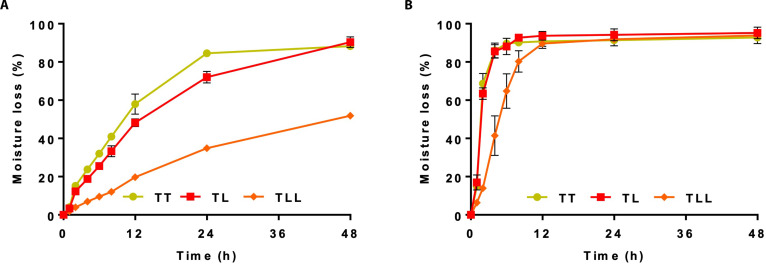
Moisture loss of TL and TLL. (A) Moisture loss at RT. (B) Moisture loss at 37 °C. Values are expressed as mean ± SD (*n* = 3).

The moisturizing effects of TL and TLL formulations were evaluated through moisture loss studies conducted at RT and 37 °C. At RT, the moisture loss rates at 48 h for the TT, TL, and TLL groups were 88.3 ± 1.4%, 90.4 ± 2.8%, and 51.9 ± 1.1%, respectively (Fig. 4A). At 37 °C, the moisture loss rates at 48 h for the TT, TL, and TLL groups were 92.8 ± 3.2%, 95.2 ± 3.0%, and 93.9 ± 1.0%, respectively (Fig. 4B). No significant difference was observed between the TT and TL groups at either temperature condition. At RT, TLL showed approximately 40% greater moisturizing efficacy compared to TL. At 37 °C, *K* and *H*_½_ were 0.4635 and 1.50 for TL, and 0.1839 and 3.77 for TLL, respectively. This shows that at 37 °C, moisture loss occurred 2.5-fold more slowly in TLL than in TL. These results indicate that TLL provides a moisturizing effect attributable to the presence of HA.

### In vitro release of TT

**Fig. 5.**
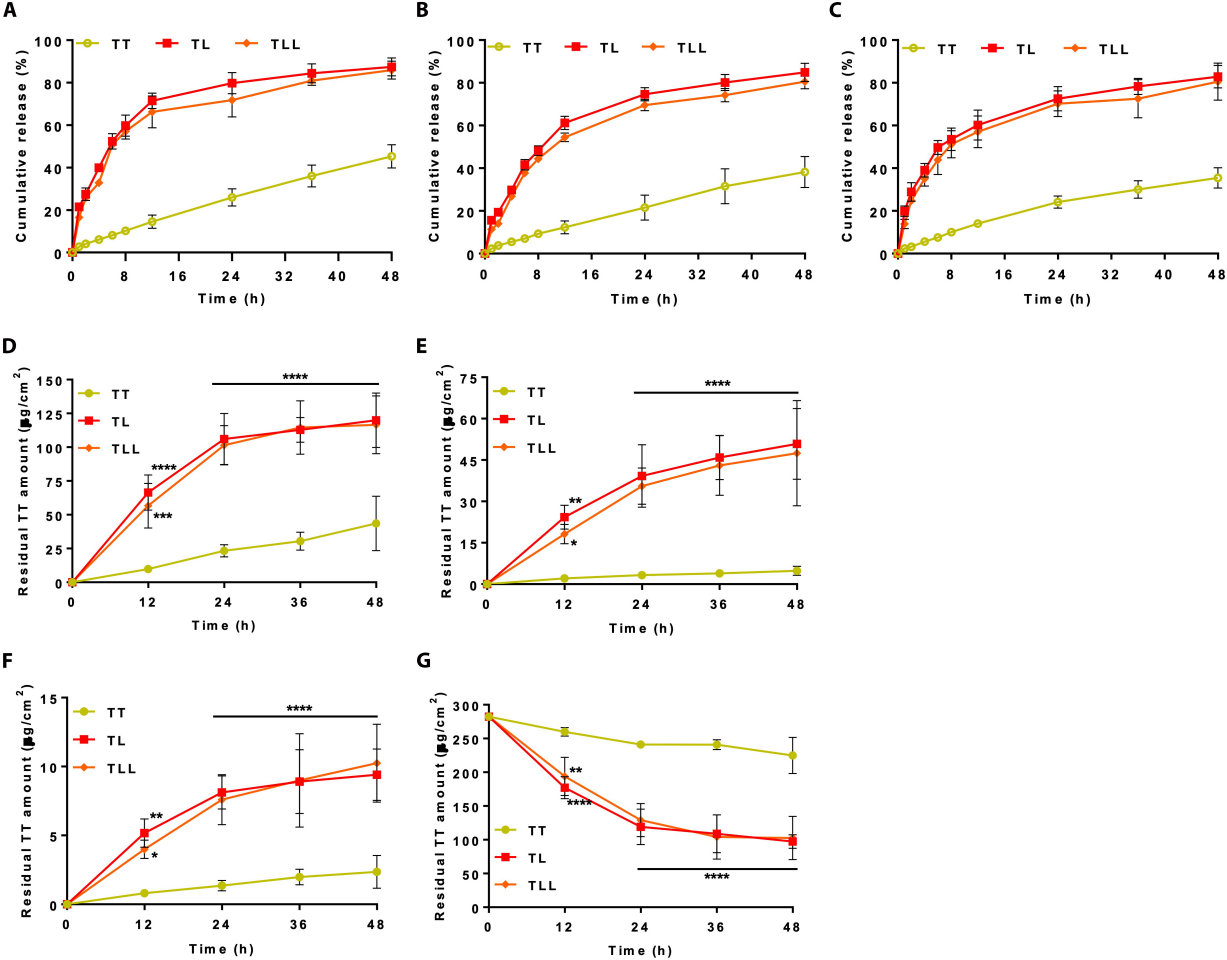
In vitro study and skin residual study. (A) In vitro release profiles in pH 5.5. (B) In vitro release profiles in pH 7.4. (C) In vitro release profiles in pH 8.5. (D) Residual TT in stratum corneum. (E) Residual TT in epidermis. (F) Residual TT in dermis. (G) Residual TT in donor compartment. Values are represented as mean ± SD (*n* = 3 or 5). *P < 0.05 versus TT, **P < 0.01 versus TT, *** *P* < 0.001 versus TT, **** *P* < 0.0001 versus TT.

The cumulative release profiles of TT, TL, and TLL formulations were evaluated at pH 5.5, pH 7.4, and pH 8.5 to evaluate their potential for the treatment of AD. pH 5.5 represents the ideal normal skin condition, pH 7.4 represents the pH of plasma and extracellular fluid, and pH 8.5 represents the AD condition mimicking a damaged skin barrier. As a low-solubility compound, the cumulative release rate of TT for 48 h reached 43.0 ± 5.5%, 38.2 ± 7.2%, and 35.5 ± 4.8% at pH 5.5, 7.4, and 8.5, respectively. In pH 5.5 buffer, the cumulative release rate of TT from TL and TLL reached 87.4 ± 4.3% and 85.9 ± 4.2% for 48 h, respectively (Fig. 5A). The cumulative release rate of TT from TL and TLL reached 84.9 ± 4.3% and 80.5 ± 3.3% for 48 h, respectively, in pH 7.4 buffer (Fig. 5B). In addition, at pH 8.5 buffer, the cumulative release percentages of TT from TL and TLL reached 82.9 ± 5.3% and 80.6 ± 8.7% for 48 h, respectively (Fig. 5C). The sustained release pattern was similar to the previously studied drug release profile of pegylated liposomes [40].

TT consistently showed low release rates at all pH levels due to its poor solubility, indicating limited therapeutic efficacy as a drug. On the other hand, the cumulative release rate of TL improved by 2.0-, 2.2-, and 2.3-fold at each respective pH level compared with TT due to the increased solubility provided by the liposome. TLL exhibited a sustained release pattern similar to that of TL. The statistical significance was not observed in the cumulative release of TT in both formulations. Additionally, the cumulative release of TT from each formulation at pH 8.5 and pH 5.5 appeared to be higher at pH 5.5 than at pH 8.5, but statistical significance was not observed. The TT formulation with enhanced solubility and sustained release is expected to be more effective for the treatment of AD than TT.

### Cell viability study

The cytotoxicity to HaCaT cells was evaluated using the MTT assay, and experiments were performed by preparing TT, BLL, TL, and TLL with the same concentration of TT (Fig. S5). As a result of observing the change in cell viability according to the concentration of TT in HaCaT cells, TT showed relatively low cytotoxicity at low concentrations, but the cell viability decreased as the concentration increased. The median inhibitory concentration (IC_50_) values of TT were 40.3 μg/ml at 24 h and 12.3 μg/ml at 48 h, confirming the toxicity of TT to HaCaT cells. On the other hand, the IC_50_ values of TL were 75.3 and 35.7 μg/ml at 24 and 48 h, respectively, indicating that TL had lower cytotoxicity than TT. It can be confirmed that liposomes can reduce the cytotoxic effect by controlling the release rate of TT. The IC_50_ values of TLL were 103.3 and 36.7 μg/ml at 24 and 48 h, respectively. Similar to TL, TLL exhibited lower cytotoxicity compared to free TT. The IC_50_ values of BLL were 405.1 and 319.1 μg/ml at 24 and 48 h, respectively. BLL exhibited high IC₅₀ values at both 24 and 48 h. Moreover, a substance is generally considered cytotoxic when it reduces cell viability by more than 70%. However, BLL maintained cell viability above 60% at all tested concentrations and time points, indicating low cytotoxicity. The cytotoxicity results indicated that BLL exhibited low toxicity to HaCaT cells at both time points. This suggests that the formulation is safe and possesses excellent biocompatibility. The 24-h IC_50_ value of TLL was higher than that of TL, which may be attributed to the lotion formulation. Consequently, the 48-h IC_50_ value of TLL was similar to that of TL. This could be due to the absence of a significant difference in the cumulative release rate between TL and TLL at 48 h.

### Skin permeation and residual studies

The skin permeation and residual studies were conducted to evaluate the amount of TT that reaches the target skin tissue and the potential for systemic exposure-related adverse effects. In the 48-h skin permeation study, no TT permeation was observed in any of the groups (TT, TL, or TLL). From 8 to 48 h, the residual TT amount in the skin differed significantly between the TT and the TT-loaded formulations. However, there was no significant difference between TL and TLL, which may be attributed to the statistically similar release rates of the 2 formulations. The residual TT amounts in the stratum corneum at 48 h were 43.6 ± 20.2, 119.9 ± 20.2, and 116.5 ± 21.4 μg/cm^2^ for free TT, TL, and TLL, respectively (Fig. 5D). In the epidermis, the residual TT amounts were 4.8 ± 1.7, 50.9 ± 12.8, and 47.5 ± 19.1 μg/cm^2^, respectively (Fig. 5E). In the dermis, the residual TT amounts were 2.4 ± 1.2, 9.4 ± 1.9, and 10.2 ± 2.8 μg/cm^2^, respectively (Fig. 5F). Compared to TT, TL exhibited 2.8-, 10.5-, and 4.0-fold higher residual TT in the stratum corneum, epidermis, and dermis, respectively. Similarly, TLL showed 2.7-, 9.8-, and 4.3-fold higher residual TT in the respective layers. TT that failed to permeate the skin was detected in the donor compartment. The residual TT amounts in the donor compartment at 48 h were 225.0 ± 26.8, 97.4 ± 10.1, and 102.5 ± 32.2 μg/cm^2^ for TT, TL, and TLL, respectively, indicating that TT exhibited approximately 2.3- and 2.2-fold higher residue in the donor compartment compared to TL and TLL, respectively (Fig. 5G).

In this study, the TT formulation enhanced TT delivery more effectively than TT, without detectable permeation, showing greater accumulation in the stratum corneum, epidermis, and dermis. These results indicate that the liposomal formulations were associated with enhanced skin residual TT amounts without detectable transdermal permeation under the present experimental conditions. Previous studies have reported that liposomal formulations improve penetration into the epidermis and dermis, which is attributed to the unique properties of liposomes [41]. Additionally, TLL showed no significant difference in residual TT amounts compared to TL. This indicates that TLL delivers TT to the skin at a rate similar to that of TL, and that the lotion formulation composed of HA and Carbopol 971 NF does not significantly affect TT penetration into the skin. Although these data demonstrate improved cutaneous retention, they do not by themselves define the precise retention mechanism. Additional studies such as penetration-depth imaging, follicular localization, transepidermal water loss assessment, and rheological characterization will be required to further clarify how the nanosystem and semisolid vehicle influence skin deposition behavior. During lotion formulation screening, the final selected TLL showed an apparent viscosity of 723.00 ± 1.28 cP, which supports the plausibility that the HA/Carbopol 971 NF-based semisolid vehicle contributed to local residence and dispersion stability. However, because this value was obtained from formulation screening rather than from comprehensive rheological characterization, it should be interpreted as supportive rather than definitive mechanistic evidence. Although a direct causal linkage between each formulation variable and biological outcome was not experimentally isolated in this study, the optimized nanosystem characterized by small particle size, narrow size distribution, and high EE was associated with enhanced skin retention, lower in vitro cytotoxicity, and improved therapeutic performance in vivo.

### In vivo study

#### Ear thickness and AD scoring

**Fig. 6.**
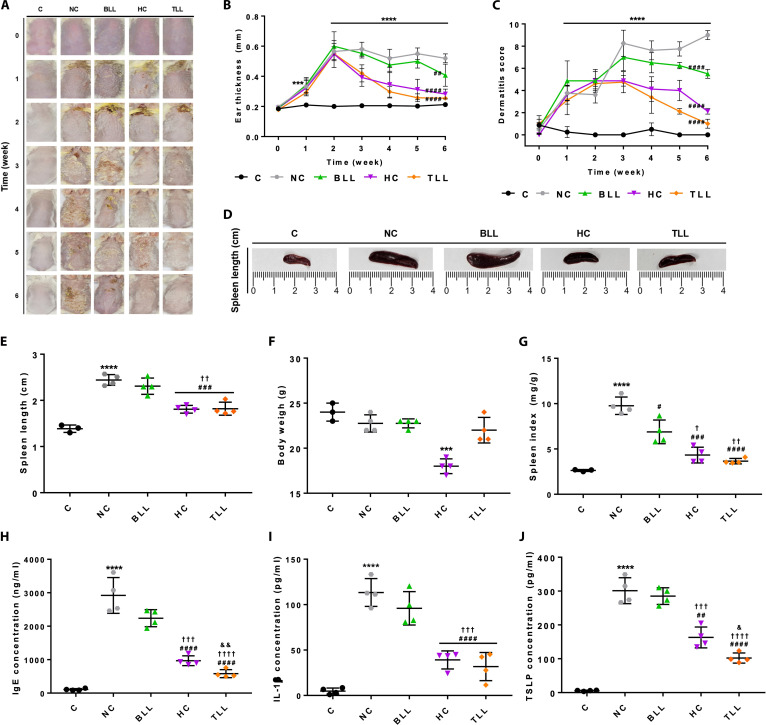
AD-induced mouse model study. (A) Representative images of the dorsal area in AD-induced mice for 6 weeks. (B) Ear thickness. (C) Dermatitis score. (D) Representative spleen images. (E) Spleen length (cm). (F) Body weight (g). (G) Spleen index (spleen weight/body weight, mg/g). (H) Concentration of IgE. (I) Concentration of IL-1β. (J) Concentration of TSLP. Values are represented as mean ± SD (*n* = 3 to 4). ^***^P < 0.001 versus C, ^****^P < 0.0001 versus C, ^##^*P* < 0.01 versus NC, ^#^*P* < 0.05 versus NC, ^###^*P* < 0.001 versus NC, ^####^*P* < 0.0001 versus NC, ^†^*P* < 0.05 versus BLL, ^††^*P* < 0.01 versus BLL, ^†††^*P* < 0.001 versus BLL, ^††††^*P* < 0.0001 versus BLL, ^&^*P* < 0.05 versus HC, ^&&^*P* < 0.01 versus HC. If it is not statistically significant, it is indicated as ns.

The ear thickness of the C, NC, BLL, HC, and TLL groups was evaluated. Figure 6B shows the ear thickness measurements of AD model mice for 6 weeks. Ear thickness increases as AD progresses. The C group was not induced with AD, and its ear thickness remained close to 0.2 mm for 6 weeks. The ear thickness at week 6 was 0.21 ± 0.01 mm. The NC group was treated with PBS after AD was induced using DNCB. In this group, ear thickness increased to approximately 0.55 mm during 2 weeks of application with 1.0% DNCB and remained approximately 0.55 mm during 4 weeks of application with 0.5% DNCB. The ear thickness at week 6 was 0.52 ± 0.03 mm (Fig. S6A). BLL group is a blank formulation without TT from TLL. Ear thickness in this group gradually decreased for 6 weeks. Until week 5, no significant difference was observed compared to the NC group. However, a statistically significant difference was observed at week 6. The ear thickness at week 6 was 0.41 ± 0.07 mm (Fig. S6B). This improvement in ear thickness may be attributed to the moisturizing effect of HA present in the formulation. The HC group was treated with a topical steroid. HC is known as a mild topical steroid. In this group, ear thickness showed a significant difference from the NC group immediately after treatment. Ear thickness began to decrease from week 3 and continued to decline steadily until week 6, with a final measurement of 0.28 ± 0.03 mm (Fig. S6C). The TLL group was treated with the final formulation. Similar to the HC group, a significant difference in ear thickness was observed compared to the NC group immediately after treatment. The ear thickness began to decrease from week 3 and continued to decline until week 6, with a final measurement of 0.26 ± 0.01 mm (Fig. S6D). There was no statistically significant difference between the HC and TLL groups. This suggests that TLL exhibits an equivalent effect to HC in improving ear thickness in the AD mouse model.

The dermatitis scores of the C, NC, BLL, HC, and TLL groups were evaluated. Figure 6A shows representative dorsal lesion images of AD-induced mice in each group over 6 weeks. Figure 6C shows the dermatitis score measurements of AD-induced mice over 6 weeks. As AD was not induced in the C group, the dermatitis score remained near 0 throughout the period. In contrast, the NC group exhibited erythema, scarring, and edema in the lesion area due to AD induction, with a progressive increase in the dermatitis score over 6 weeks. The score reached 9.0 ± 0.4 at week 6 (Fig. S6A). The BLL, HC, and TLL groups were treated with their respective formulations applied to the dorsal AD lesion areas, resulting in improved skin conditions compared to the NC group. The BLL group exhibited a gradual decrease in dermatitis scores. No significant difference was observed compared to the NC group until week 4. However, a statistically significant reduction was noted from week 5 onward. At week 6, the dermatitis score in the BLL group was 5.5 ± 0.4 (Fig. S7B). In the HC group, the dermatitis score began to decrease immediately after treatment. A statistically significant and continuous reduction was observed from week 3 to week 6 compared to the NC group. At week 6, the dermatitis score in the HC group was 2.1 ± 0.3, representing a 76.7% reduction compared to the NC group and a 1.97-fold greater reduction in dermatitis score than that achieved by the BLL group (Fig. S7C). The TLL group showed a reduction in dermatitis score immediately after treatment, similar to the HC group. A statistically significant and continuous decrease was observed from week 3 to week 6 compared to the NC group. At week 6, the dermatitis score in the TLL group was 1.0 ± 0.4, representing an 88.9% reduction compared to the NC group. Moreover, the TLL group exhibited a 2.30- and 1.16-fold greater reduction in dermatitis score than the BLL and HC groups, respectively (Fig. S7D). A statistically significant difference was observed between the TLL and HC groups, suggesting that TLL is more effective than HC in improving the dermatitis score in AD-induced mice. The partial improvement observed in the BLL group indicates that the HA/Carbopol-based vehicle itself contributed to symptom relief, likely through moisturization and barrier-supportive effects. Therefore, the current study does not claim that liposomal encapsulation was the sole determinant of efficacy. Rather, the superior performance of TLL over BLL suggests that incorporation of TT into the optimized liposomal system provided an additional therapeutic benefit beyond that of the blank vehicle.

#### IgE concentration, spleen length, and spleen index

An increase in blood IgE concentration is a characteristic feature of AD. The IgE concentration was measured using a mouse IgE ELISA kit. The IgE concentrations in the C, NC, BLL, HC, and TLL groups were 107.2 ± 28.3, 2,920.5 ± 535.1, 2,239.7 ± 254.4, 968.0 ± 147.9, and 581.6 ± 119.5 ng/ml, respectively (Fig. 6H). No significant difference in IgE concentration was observed between the NC and BLL groups. Compared to the NC group, the HC and TLL groups showed 66.8% and 80.1% reductions in IgE concentration, respectively. Consequently, TLL reduced IgE concentration by 13.3% more than HC when compared to the NC group. These results suggest that the TLL group is more effective than the HC group in reducing IgE concentration in AD-induced mice.

In AD or psoriasis models, an increase in spleen length and weight is generally used as an indicator of inflammatory and immune response activation. A reduction in spleen length and weight following treatment can be interpreted as evidence of suppressed inflammatory responses. Therefore, it indirectly suggests a therapeutic or alleviating effect on atopic symptoms. Representative spleen images for each group are presented in Fig. 6D. The spleen lengths in the C, NC, BLL, HC, and TLL groups were 1.39 ± 0.08, 2.44 ± 0.12, 2.31 ± 0.18, 1.81 ± 0.08, and 1.82 ± 0.14 cm, respectively (Fig. 6E). The body weight in C, NC, BLL, HC, and TLL groups was 24.0 ± 1.0, 22.8 ± 1.0, 22.75 ± 0.5, 18.0 ± 0.8, and 22.0 ± 1.4 g, respectively (Fig. 6F). The NC, BLL, and TLL groups showed body weights comparable to that of the C group, whereas the HC group exhibited a significantly lower body weight. The spleen index in the C, NC, BLL, HC, and TLL groups was 2.63 ± 0.11, 9.77 ± 0.97, 6.89 ± 1.31, 4.33 ± 0.87, and 3.66 ± 0.29 mg/g, respectively (Fig. 6G). Statistically significant differences were observed in all formulation-treated groups compared to the NC group. The spleen index was reduced by approximately 29.5%, 55.7%, and 62.5% in the BLL, HC, and TLL groups, respectively, relative to the NC group. This indirectly indicates suppression of the inflammatory response in the formulation-treated groups. Additionally, no statistically significant difference was observed between the TLL and HC groups, suggesting that TLL exerts an effect comparable to HC in reducing spleen weight, an indicator of inflammation and immune response activation.

AD is understood as a systemic immune-mediated disorder rather than a condition limited to the skin. This is supported by alterations in both cutaneous and systemic inflammatory and immunological biomarkers [42]. IgE is an important therapeutic target for allergy and AD as it is the major mechanism for activating mast cells to release histamine [43]. Induction of AD using DNCB leads to splenomegaly, a characteristic change in the secondary lymphoid organ. This enlargement reflects the activation of systemic immune responses and, as previously mentioned, serves as an indirect indicator for evaluating inflammation resolution and immune modulation [44]. Based on the IgE concentration and spleen data results, BLL may have limited efficacy in reducing inflammation in AD. Although the IgE concentration in the BLL group was lower than that in the NC group, the difference was not statistically significant. Additionally, while the spleen index was significantly lower in the BLL group compared to the NC group, the spleen index is only an indirect marker of inflammation suppression. In contrast, both HC and TLL significantly inhibited inflammation in AD. TLL was as effective as HC in reducing the spleen index and achieved a greater reduction in IgE concentration than HC. In addition, a decrease in body weight was observed in the HC group. Weight loss is a well-known adverse effect associated with both topical and systemic administration of corticosteroids [45]. These findings indicate that TLL could be a more promising therapeutic strategy for the treatment of AD than HC.

#### IL-1β and TSLP concentration

In the AD mouse model, an increase in IL-1β concentration is predominantly observed in local tissues, but increases have also been reported in the blood [46]. The IL-1β concentrations in the C, NC, BLL, HC, and TLL groups were 4.9 ± 3.3, 113.4 ± 15.3, 95.9 ± 18.4, 39.2 ± 10.0, and 31.8 ± 15.5 pg/ml, respectively (Fig. 6I). Although the BLL group showed a lower IL-1β level than the NC group, this difference was not statistically significant. In contrast, both the HC and TLL groups demonstrated significant reductions in IL-1β levels compared to the NC and BLL groups. Specifically, the HC group achieved reductions of approximately 66.9% compared to the NC group and 56.8% compared to the BLL group, while the TLL group achieved reductions of 80.1% and 74.0% compared to the NC and BLL groups, respectively. No significant difference in IL-1β levels was observed between the HC and TLL groups.

The concentrations of TSLP in the C, NC, BLL, HC, and TLL groups were 11.2 ± 2.8, 301.2 ± 38.4, 285.1 ± 24.8, 163.0 ± 30.9, and 102.2 ± 14.9 pg/ml, respectively (Fig. 6J). In the AD-induced mouse model, an increased TSLP concentration was observed. No statistically significant difference was found between the NC and BLL groups. In contrast, the HC and TLL groups significantly reduced TSLP concentrations by 45.9% and 42.8% compared to the NC and BLL groups, respectively, for the HC group, and by 66.1% and 64.2% compared to the NC and BLL groups, respectively, for the TLL group. Notably, the TLL group reduced TSLP concentration by more than 20% compared to the HC group.

The reduction in IL-1β and TSLP in the HC group is attributed to the anti-inflammatory effects of corticosteroids [47]. The decreases in IL-1β and TSLP observed in the TLL group are consistent with the previously reported anti-inflammatory activity of TT. Together with the NF-κB p65 immunohistochemistry findings, these data suggest that the anti-inflammatory effect of TLL is associated with attenuation of NF-κB-related inflammatory signaling in lesional skin. However, direct upstream analyses of Rip2 and caspase-1 were not performed in the present study.

#### H&E and toluidine blue stain

**Fig. 7.**
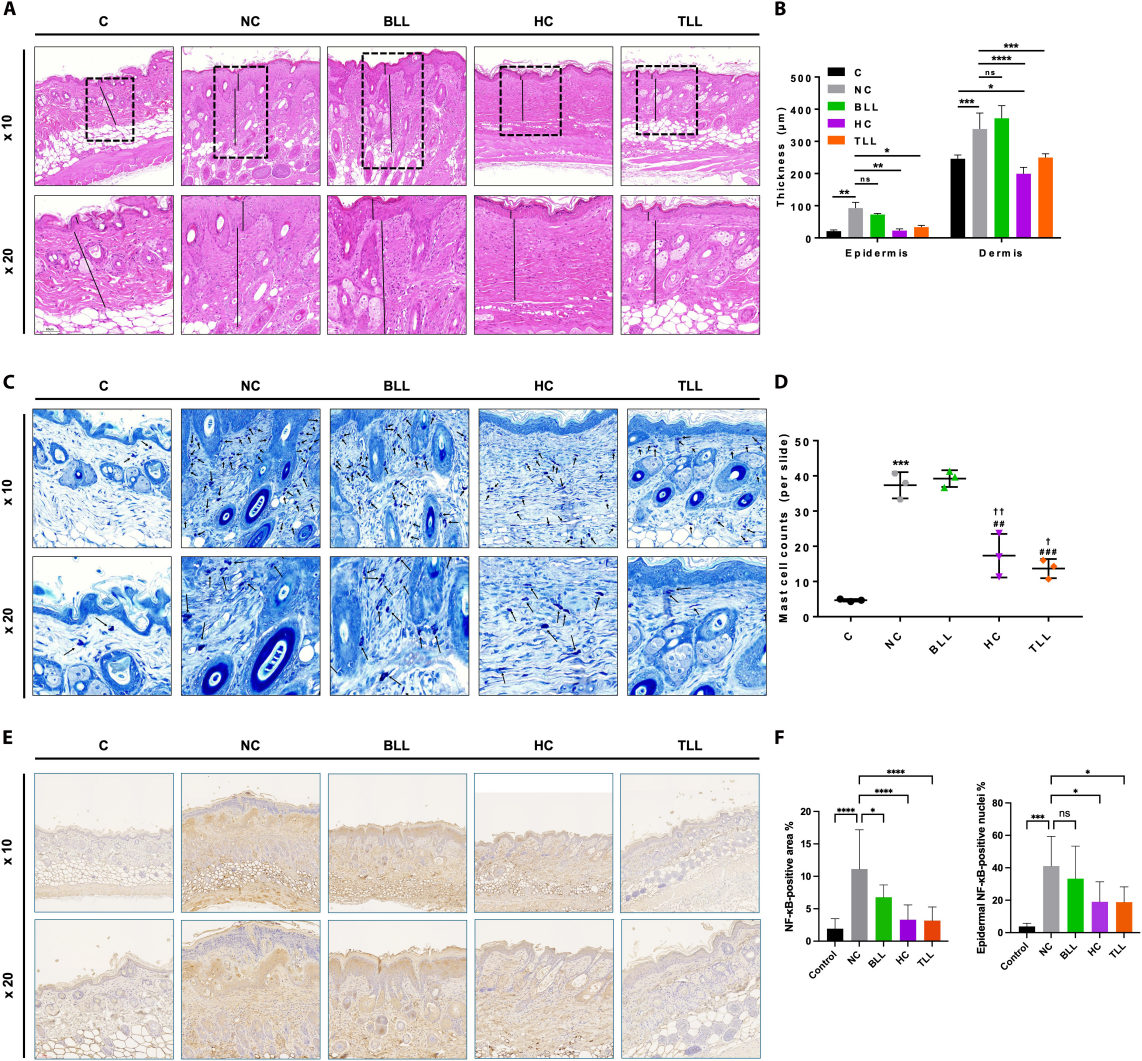
Histological and NF-κB p65 immunohistochemical analysis of skin tissue in the AD mouse model. (A) Representative H&E-stained images of each group at 10× and 20× magnification. (B) Quantification of epidermal and dermal thickness in each group. (C) Representative toluidine blue-stained images of each group at 20× and 40× magnification. (D) Mast cell counts in each group. (E) Representative NF-κB p65 immunohistochemical images of each group at 10× and 20× magnification. (F) QuPath-based quantification of DAB-positive area (%) and DAB-positive cell nuclei (%). Mice were divided into control (*n* = 3), NC (*n* = 4), HC (*n* = 4), BLL (*n* = 4), and TLL (*n* = 4) groups. Histological values are represented as mean ± SD (*n* = 3). NF-κB p65 quantification was performed on representative lesional fields (DAB-positive area, *n* = 9; DAB-positive cell nuclei, *n* = 9). *P < 0.05, **P < 0.01, ***P < 0.001, ****P < 0.0001, ^#^*P* < 0.05 versus NC, ^##^*P* < 0.01 versus NC, ^###^*P* < 0.001 versus NC, ^†^*P* < 0.05 versus BLL, ^††^*P* < 0.01 versus BLL. If there was no statistically significant difference, it is indicated as ns.

To evaluate epidermal and dermal thickness as well as immune cell infiltration in the AD mouse model, histological analyses were performed using H&E staining and toluidine blue staining (Fig. S8). The epidermal thicknesses in the C, NC, BLL, HC, and TLL groups were 21.0 ± 3.9, 92.7 ± 18.0, 72.9 ± 3.0, 22.5 ± 5.6, and 33.6 ± 5.5 μm, respectively. The dermal thicknesses were 246.2 ± 11.7, 338.5 ± 49.7, 372.0 ± 39.0, 199.3 ± 20.1, and 249.7 ± 11.7 μm, respectively (Figs. 6B and 7A). Marked thickening of both epidermis and dermis was observed in the NC and BLL groups. Compared with the NC group, the HC group showed reductions of 75.8% and 41.1% in epidermal and dermal thickness, respectively, while the TLL group exhibited reductions of 63.7% and 26.2%, respectively. The dermal thickness in the HC group was significantly lower than that of the C group.

Given that mast cell infiltration is typically triggered in AD, toluidine blue staining was performed to assess mast cell counts (Fig. 7C). The number of mast cells in the C, NC, BLL, HC, and TLL groups was 4.7 ± 0.3, 37.3 ± 3.7, 39.2 ± 2.4, 17.3 ± 6.2, and 13.7 ± 2.7, respectively (Fig. 7D). A substantial increase in mast cells was observed in the NC group following AD induction. BLL treatment did not reduce mast cell numbers, whereas both HC and TLL groups showed effective reductions. Compared with the NC group, mast cell counts were decreased by 53.6% and 63.4% in the HC and TLL groups, respectively.

H&E staining of dorsal sections revealed morphological alterations in skin structure, such as hyperplasia, following DNCB treatment [44]. Local infiltration of mast cells was confirmed by toluidine blue staining. Histamine, released by activated mast cells, allows immune cells to infiltrate into tissue by increasing the permeability of blood vessels [48]. To reduce the allergic response in AD, it is necessary to control activated mast cells. While HC effectively reduced skin thickness and mast cell counts in the AD model, adverse effects commonly associated with corticosteroid use, such as skin atrophy and reduction of subcutaneous fat, were observed. In contrast, TLL achieved comparable therapeutic outcomes without observable adverse effects. These findings suggest that TLL may offer a safer alternative for reducing AD-associated skin thickening and mast cell infiltration.

#### NF-κB p65 immunohistochemistry

To further evaluate inflammation-associated NF-κB activity in lesional skin, NF-κB p65 immunohistochemistry was performed on dorsal skin sections and quantified by QuPath-based digital image analysis (Fig. 7E and F). Minimal NF-κB p65 staining was observed in the control group, whereas the NC group showed markedly increased NF-κB p65 immunoreactivity following DNCB induction. The DAB-positive area (%) in the control, NC, BLL, HC, and TLL groups was 3.80 ± 1.99, 41.12 ± 18.26, 33.29 ± 20.07, 19.02 ± 12.36, and 18.84 ± 9.37, respectively. In addition, the DAB-positive cell nuclei (%) were 1.92 ± 1.55, 11.12 ± 6.06, 6.77 ± 1.90, 3.31 ± 2.28, and 3.18 ± 2.09, respectively. Compared with the NC group, TLL reduced the DAB-positive area by approximately 54.2% and reduced DAB-positive cell nuclei by approximately 71.4%, whereas HC reduced these parameters by approximately 53.7% and 70.2%, respectively. The BLL group showed only partial attenuation of NF-κB p65 staining. Because nuclear localization of NF-κB p65 is a key indicator of NF-κB activation, the marked reduction in DAB-positive nuclei in the TLL group supports suppression of lesion-associated NF-κB activation in vivo. These findings are consistent with the reductions in IL-1β and TSLP observed in the TLL group and support the interpretation that TLL attenuated NF-κB-associated inflammatory signaling in skin lesions.

## Conclusion

AD is a chronic, inflammatory, and relapsing skin disease that requires continuous management and treatment. Topical corticosteroids have been widely used as an effective therapy for AD. Their therapeutic effects are primarily attributed to anti-inflammatory and immunosuppressive mechanisms that suppress inflammation-associated skin disorders such as AD. However, long-term use of topical corticosteroids is associated with various adverse effects, including skin atrophy, depigmentation, and tachyphylaxis. In infants, prolonged use may lead to systemic side effects such as growth retardation and osteoporosis. In addition to topical corticosteroids, topical calcineurin inhibitors, biologic agents, and JAK inhibitors have been introduced for the treatment of AD. Nonetheless, their use is often restricted by adverse effects and high treatment costs, thereby limiting their long-term applicability in the management of AD [14–16]. Therefore, there is an unmet need for the development of new therapeutic agents that can provide essential efficacy in AD treatment without causing adverse effects.

TT is a compound with diverse pharmacological potential, including therapeutic efficacy for AD. However, its poor aqueous solubility limits its therapeutic application. Liposomes are capable of encapsulating poorly soluble drugs and can effectively deliver drugs to the skin while reducing systemic side effects. In this study, the characteristics of TT and liposomes were utilized, and the developed formulation was evaluated. After the TL was optimized, a lotion was formulated to improve the inherent low stability of liposomes. The physicochemical properties, in vitro efficacy, and in vivo efficacy of the liposome and the liposome lotion formulation were evaluated.

TL was optimized by a BBD with a Soy-PC amount of 26.9 μmol, a Chol/Soy-PC ratio of 31.6%, and a DMG-PEG/Soy-PC ratio of 11.4%. The optimized TL exhibited a small droplet size of 80.8 ± 1.3 nm, a low PDI of 0.178 ± 0.002, and a high EE of 72.1 ± 2.6%. DSC and FT-IR spectra indicated that TL was amorphous and that TT was successfully encapsulated in the liposomes. However, the stability of TL was limited to 6 d at 4 °C. In contrast, TLL, due to the incorporation of Carbopol 971 NF and HA, remained stable for up to 3 weeks at 4 °C and showed improved moisturizing effects. In vitro release studies showed that both TL and TLL exhibited sustained release profiles at pH 5.5, 7.4, and 8.5, with higher cumulative release rates compared to TT. Cytotoxicity studies using HaCaT cells showed that TL and TLL had lower cytotoxicity than TT. In skin permeation and residual studies, both TL and TLL delivered a greater amount of TT retained in the skin without transdermal penetration, compared to TT.

To confirm the in vivo effect of TLL, the DNCB-induced AD mouse model was used, which induces dermatitis within a relatively short period and allows for easy visual observation of skin symptoms such as lichenification and blisters. In this study, topical application of DNCB to the dorsal skin of mice induced various clinical symptoms of AD, and the skin lesions were similar to those observed in patients with AD. Epidermal thickening was observed in the skin lesions of DNCB-treated mice, and this DNCB-induced epidermal thickening was markedly reduced in both the HC and TLL groups. However, skin atrophy, a typical adverse effect of topical corticosteroid treatment, was observed in the HC group. In contrast, although epidermal thickening was significantly reduced in the TLL group, no skin atrophy was observed. Moreover, the TLL group showed reductions in ear thickness, spleen index, and IL-1β concentrations comparable to those in the HC group while showing greater reductions in dermatitis scores, IgE concentrations, and TSLP concentrations than the HC group.

Topical and systemic administration of corticosteroids has been reported to reduce body weight in experimental animals, and such weight loss is generally recognized as one of the side effects resulting from immunosuppression [45]. In the present study, TLL showed superior therapeutic efficacy against AD in the DNCB-induced AD mouse model compared to HC. Treatment with HC led to a reduction in body weight and was associated with the occurrence of skin atrophy. In contrast, TLL treatment did not affect body weight and no skin atrophy was observed. These results suggest that TLL provides AD therapeutic efficacy equal to or greater than that of HC without the associated adverse effects.

Collectively, TLL improved the limited physicochemical stability and moisturizing performance of the optimized TL while maintaining similar release behavior and skin retention, thereby enabling effective topical delivery of TT.

In the DNCB-induced AD mouse model, TLL showed therapeutic efficacy comparable to or greater than that of the clinically used comparator HC, without observable adverse effects such as body weight loss or skin atrophy under the present experimental conditions. These findings suggest that TLL may serve as a promising corticosteroid-free topical formulation strategy for the treatment of AD. The significance of this study lies in formulation engineering and therapeutic application of a TT-loaded nanoliposomal lotion, rather than in the introduction of a new material architecture. Although the present study did not include a free TT-mixed lotion group, the comparative results with BLL indicate that the lotion base contributed partially to symptom improvement, whereas TT-loaded liposomal lotion produced a greater therapeutic effect. Several limitations of this study should be acknowledged. First, the animal study was designed as an exploratory efficacy study with a relatively small group size and a single TT dose level. Second, although TLL was monitored for 4 weeks at 4 °C, physicochemical stability based on droplet size, PDI, and EE was maintained for approximately 3 weeks under the tested condition. Third, the current study did not include a free TT-mixed lotion group; therefore, the respective contributions of liposomal encapsulation and the semisolid vehicle could not be fully separated. Fourth, while the anti-inflammatory findings were supported by IL-1β and TSLP measurements together with NF-κB p65 immunohistochemistry, direct mechanistic analyses of Rip2 and caspase-1, as well as protein- or RNA-based pathway analyses, were not performed in the present study. Therefore, the current data support attenuation of NF-κB-associated inflammatory signaling in vivo, but do not by themselves establish direct inhibition of the entire Rip2/caspase-1/NF-κB axis. Accordingly, larger-scale studies including dose–response evaluation, extended stability testing, quantitative skin safety assessment, and additional mechanistic investigations are warranted.

## Data Availability

Data will be made available on request.

## Supplementary Materials

Supplementary 1 Figs. S1 to S8 Tables S1 to S3 References [49, 50]
