# Supplementary material for: Tryptanthrin Nanoliposomal Lotion as a Corticosteroid-Free Strategy for Safe and Effective Treatment of Atopic Dermatitis
Source: Biomater Res. 2026 Jul 24;30:0384. doi: 10.34133/bmr.0384 (PMC13396491; doi:10.34133/bmr.0384)
Supplement: Supplementary 1 — Figs. S1 to S8 Tables S1 to S3 References [49, 50] [file bmr.0384.f1.doc]

SUPPLEMENTARY MATERIALS

**METHOD**

**CELL STUDY**

**Cell culture**

HaCaT cells, a spontaneously immortalized human keratinocyte cell line that developed through a long-term culture of normal human adult skin keratinocytes at reduced calcium concentration and elevated temperature, were obtained from Chosun University (Gwangju, Republic of Korea) [49]. HaCaT cells were cultured at 37°C in 5.0% CO_2_ in DMEM containing 10.0% v/v FBS and 1.0% v/v penicillin-streptomycin.

**Cell viability assay**

HaCaT cells were seeded at a density of 2 × 10^4^ in 96-well plates before 24 h at 37℃, 5.0% CO2 incubator. Free TT was dissolved in DMSO and then diluted with DMEM to prepare various concentrations. Also, BL, TL, and TLL were diluted with DMEM to prepare various concentrations of TT and treated in the cells. After incubation for 24 h and 48 h, 30 μL MTT solution (5 mg/mL) was added to each well and then, incubated for 3 h. After incubation, the medium was eliminated, and 200 μL DMSO was added. Absorbance was measured at 565 nm using a microplate reader (Multiskan sky high, Thermo Fisher Scientific, Waltham, MA, USA). The cell viability was calculated by the following equation.

*Cell viability (%) = ODsample/ODcontrol × 100*

In this equation, ODsample is the absorbance value of cells treated with test different formulations, and ODcontrol is the absorbance value of the cells cultured with the DMEM. All experiments were performed in 5 replicates (n=5).

**RESULT AND DISCUSSION**

**CELL VIABILITY STUDY**

The cytotoxicity to HaCaT cells was evaluated using the MTT assay, and experiments were performed by preparing TT, BLL, TL, and TLL with the same concentration of TT (**Fig. S5**). As a result of observing the change in cell viability according to the concentration of TT in HaCaT cells, TT showed relatively low cytotoxicity at low concentrations, but the cell viability decreased as the concentration increased. The IC_50_ values of TT were 40.3 μg/mL at 24 h and 12.3 μg/mL at 48 h, confirming the toxicity of TT to HaCaT cells. On the other hand, the IC_50_ values of TL were 75.3 μg/mL and 35.7 μg/mL at 24 h and 48 h, respectively, indicating that TL had lower cytotoxicity than TT. It can be confirmed that liposomes can reduce the cytotoxic effect by controlling the release rate of TT. The IC_50_ values of TLL were 103.3 μg/mL and 36.7 μg/mL at 24 h and 48 h, respectively. Similar to TL and TLL exhibited lower cytotoxicity compared to free TT. The IC50 values of BLL were 405.1 μg/mL and 319.1 μg/mL at 24 h and 48 h, respectively. BLL exhibited high IC₅₀ values at both 24 h and 48 h. Moreover, a substance is generally considered cytotoxic when it reduces cell viability by more than 70% [50]. However, BLL maintained cell viability above 60% at all tested concentrations and time points, indicating low cytotoxicity. The cytotoxicity results indicated that BLL exhibited low toxicity to HaCaT cells at both time points. This suggests that the formulation is safe and possesses excellent biocompatibility. The 24 h IC_50_ value of TLL was higher than that of TL, which may be attributed to the lotion formulation. Consequently, the 48 h IC_50_ value of TLL was similar to that of TL. This could be due to the absence of a significant difference in the cumulative release rate between TL and TLL at 48 h.

**Table S1.** The experimental composition and observed responses of TL (mean ± SD; n=3)

| Run | **Factors** | | | | **Responses** | | |
| --- | --- | --- | --- | --- | --- | --- | --- |
|  | X_1_ | X_2_ | X_3_ | Y_1_ | | Y_2_ | Y_3_ |
|  | Soy-PC amount (µmol) | Chol / Soy-PC ratio (%) | DMG-PEG/Soy-PC ratio (%) | Droplet size (nm) | | PDI | EE (%) |
| 1 | 25 | 25 | 11.25 | 81.5 ± 0.5 | | 0.195 ± 0.022 | 75.6 ± 2.2 |
| 2 | 40 | 25 | 20 | 72.8 ± 0.8 | | 0.169 ± 0.006 | 58.3 ± 0.1 |
| 3 | 40 | 0 | 11.25 | 80.8 ± 2.5 | | 0.199 ± 0.036 | 47.6 ± 0.3 |
| 4 | 10 | 25 | 2.5 | 68.8 ± 1.8 | | 0.280 ± 0.011 | 51.3 ± 0.3 |
| 5 | 10 | 0 | 11.25 | 83.4 ± 1.5 | | 0.326 ± 0.014 | 36.3 ± 4.4 |
| 6 | 25 | 25 | 11.25 | 81.8 ± 1.1 | | 0.196 ± 0.011 | 73.4 ± 3.7 |
| 7 | 10 | 25 | 20 | 79.3 ± 3.5 | | 0.280 ± 0.005 | 63.6 ± 3.9 |
| 8 | 40 | 25 | 2.5 | 103.8 ± 1.0 | | 0.227 ± 0.008 | 69.5 ± 2.2 |
| 9 | 25 | 50 | 2.5 | 94.5 ± 0.7 | | 0.188 ± 0.005 | 64.6 ± 3.8 |
| 10 | 25 | 25 | 11.25 | 82.4 ± 1.0 | | 0.196 ± 0.008 | 75.1 ± 1.4 |
| 11 | 40 | 50 | 11.25 | 105.4 ± 1.0 | | 0.214 ± 0.022 | 54.7 ± 2.6 |
| 12 | 25 | 0 | 20 | 75.2 ± 1.2 | | 0.188 ± 0.019 | 48.2 ± 0.2 |
| 13 | 25 | 25 | 11.25 | 82.1 ± 0.6 | | 0.195 ± 0.029 | 73.7 ± 2.7 |
| 14 | 25 | 50 | 20 | 81.6 ± 1.0 | | 0.182 ± 0.007 | 66.7 ± 1.0 |
| 15 | 25 | 0 | 2.5 | 84.8 ± 0.9 | | 0.242 ± 0.024 | 56.2 ± 0.5 |
| 16 | 25 | 25 | 11.25 | 80.9 ± 1.0 | | 0.196 ± 0.024 | 74.1 ± 0.5 |
| 17 | 10 | 50 | 11.25 | 73.0 ± 2.3 | | 0.251 ± 0.006 | 58.0 ± 4.9 |

**Table S2. Summary of model fitting and statistical analysis**

| **Responses** | **Suggested Model** | **Model *p*-value** | **Lack of fit**  ***p*-value** | **R^2^** | **Adjusted R^2^** | **Predicted R^2^** | **Adequate Precision** |
| --- | --- | --- | --- | --- | --- | --- | --- |
| Y_1_ | Quadratic | < 0.0001 | 0.3783 | 0.9985 | 0.9966 | 0.9870 | 82.9617 |
| Y_2_ | Quadratic | < 0.0001 | 0.6716 | 0.9999 | 0.9999 | 0.9997 | 416.0444 |
| Y_3_ | Quadratic | < 0.0001 | 0.1126 | 0.9939 | 0.9860 | 0.9249 | 36.6717 |

**Table S3.** Coefficient equations of responses according to the level of factors

| **Response** | **Coefficient equations** |
| --- | --- |
| Y_1_ | 81.74 + 17.29 X_1_ + 3.79 X_2_ – 5.38 X_3_ + 8.75 X_1_X_2_ – 10.38 X_1_X_3_ – 0.83 X_2_X_3_ + 0.53 X_1_^2^ + 3.38 X_2_^2^ – 1.09 X_3_^2^ |
| Y_2_ | 0.1956 – 0.0410 X_1_ - 0.0150 X_2_ – 0.0148 X_3_ + 0.0225 X_1_X_2_ – 0.0145 X_1_X_3_ + 0.0120 X_2_X_3_ + 0.0455 X_1_^2^ + 0.0064 X_2_^2^ – 0.0020 X_3_^2^ |
| Y_3_ | 74.12 + 2.61 X_1_ + 6.96 X_2_ – 0.62 X_3_ – 3.65 X_1_X_2_ – 5.89 X_1_X_3_ + 2.35 X_2_X_3_ – 11.60 X_1_^2^ – 13.37 X_2_^2^ – 1.83 X_3_^2^ |


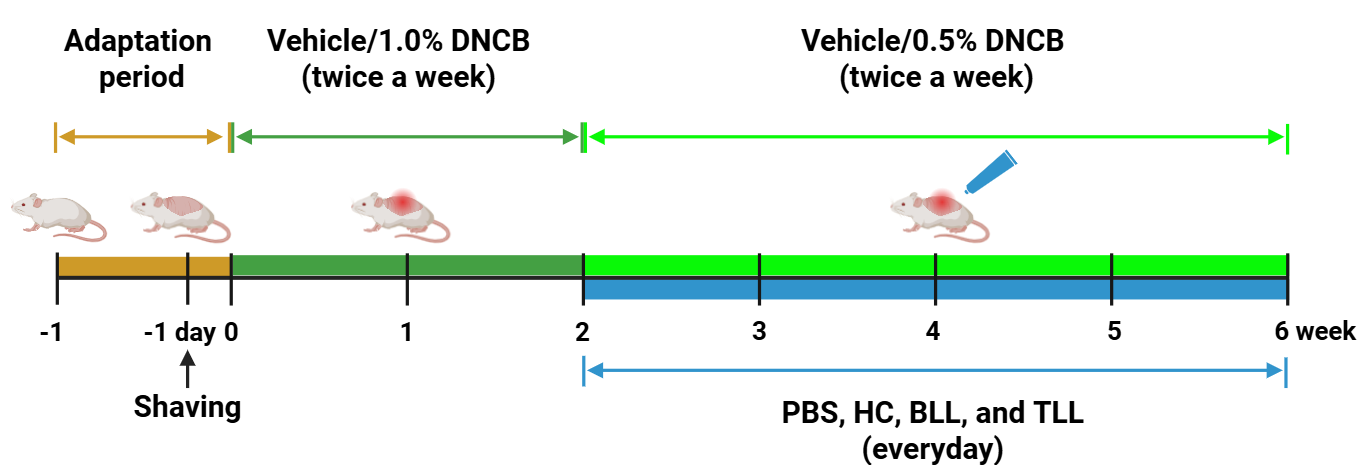


**Fig. S1.** Time regime of the DNCB-induced AD model using BALB/c mice.


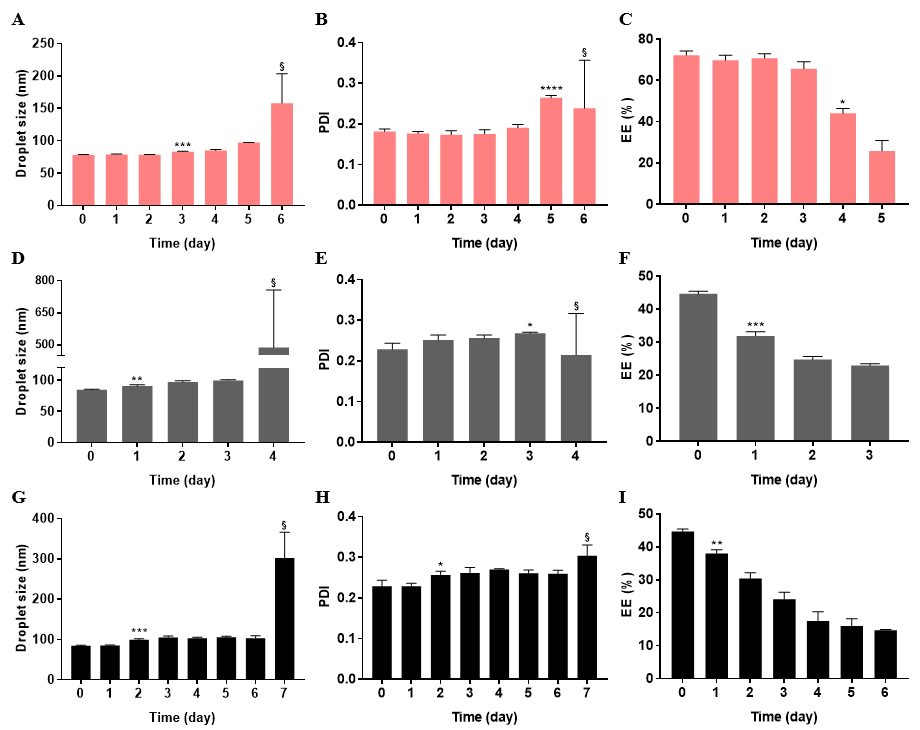


**Fig. S2.** Stability of TL (RT) and TL^-PEG^ (RT and 4ºC). (A) Droplet size of TL at RT; (B) PDI of TL at RT; (C) EE of TL at RT; (D) Droplet size of TL^-PEG^ at RT; (E) PDI of TL^-PEG^ at RT; (F) EE of TL^-PEG^ at RT; (G) Droplet size of TL^-PEG^ at 4ºC; (H) PDI of TL^-PEG^ at 4ºC; (I) EE of TL^-PEG^ at 4ºC. Liposome aggregation is indicated by the symbol §. Values are expressed as mean ± SD (n=3). **P <* 0.05 vs 0 day*,* ** *P* < 0.01 vs 0 day, *** *P* < 0.001 vs 0 day, **** *P* < 0.0001 vs 0 day.


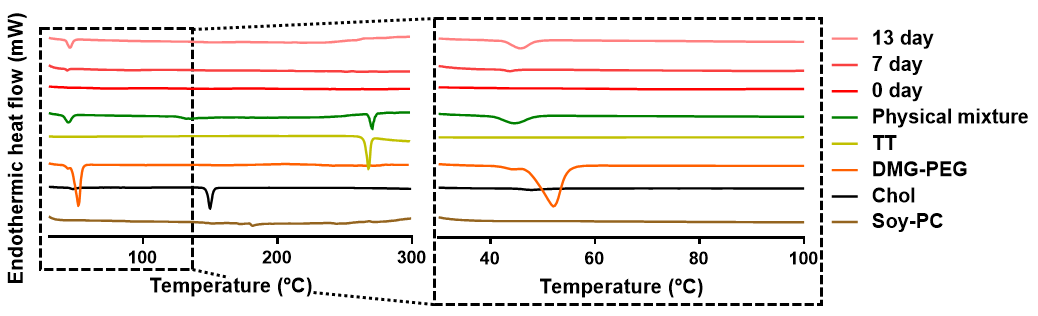


**Fig. S3.** DSC thermogram of Soy-PC, Chol, DMG-PEG, TT, physical mixture, and TL (0, 7, 13 days at 4ºC). The boxed region presents an enlarged view of the 30–100°C temperature range.


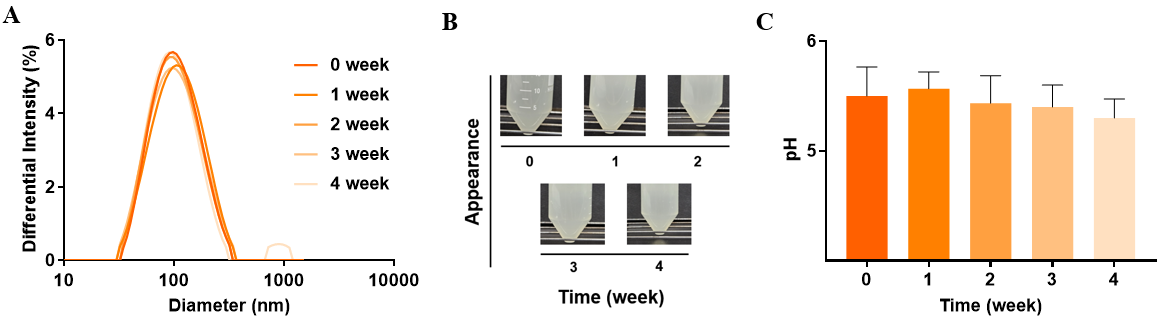


**Fig. S4.** Stability of TLL at 4ºC. (A) Droplet size distribution; (B) Appearance; (C) pH.


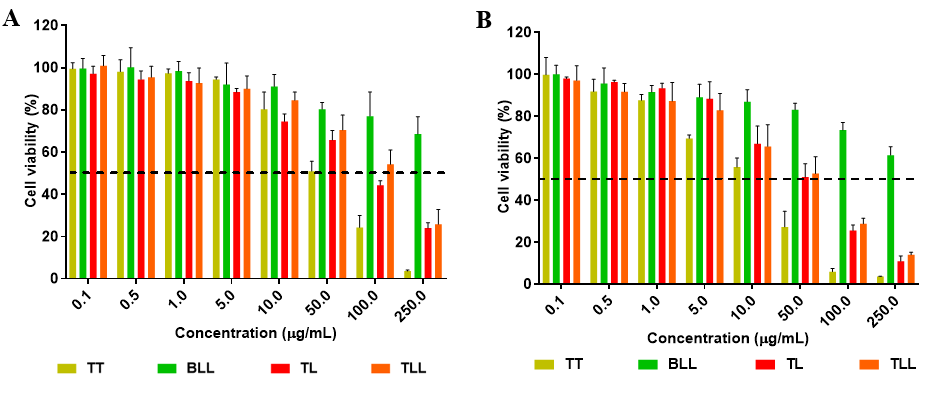


**Fig. S5.** Cell viability of HaCaT cells by TT, BLL, TL, and TLL for (A) 24 h and (B) 48 h. Values are represented as mean ± SD (n=5).


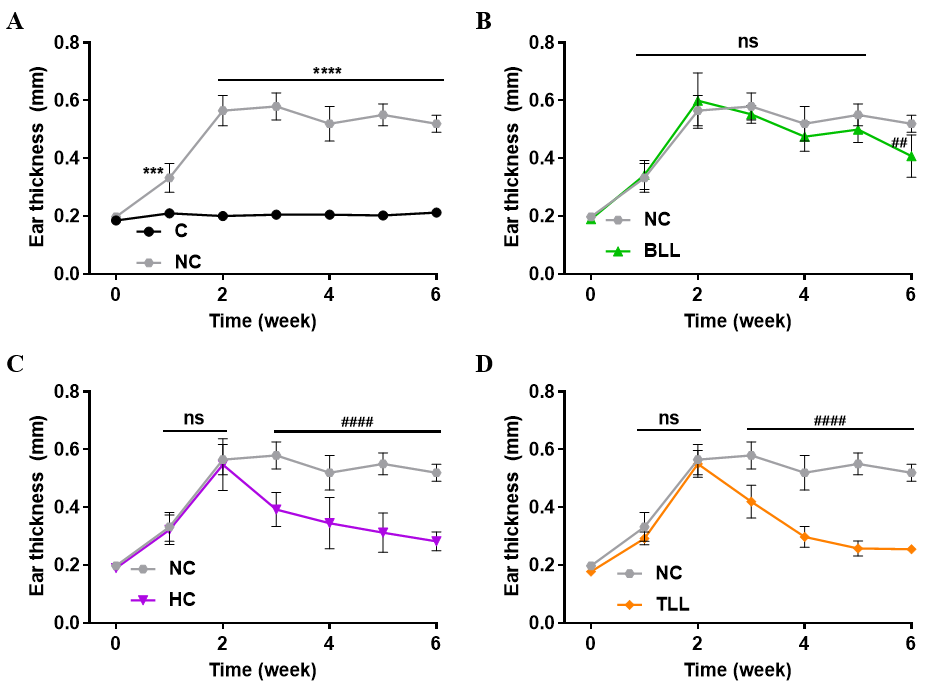


**Fig. S6.** Ear thickness in AD-induced mice over 6 weeks. (A) Comparison between C and NC groups; (B) Comparison between BLL and NC groups; (C) Comparison between HC and NC groups; (D) Comparison between TLL and NC groups. Values are represented as mean ± SD (n=4). ****P* < 0.001 vs C, **** *P* < 0.0001 vs C, ^##^ *P* < 0.01 vs NC, ^####^ *P* < 0.0001 vs NC. If it is not statistically significant, it is indicated as ns.


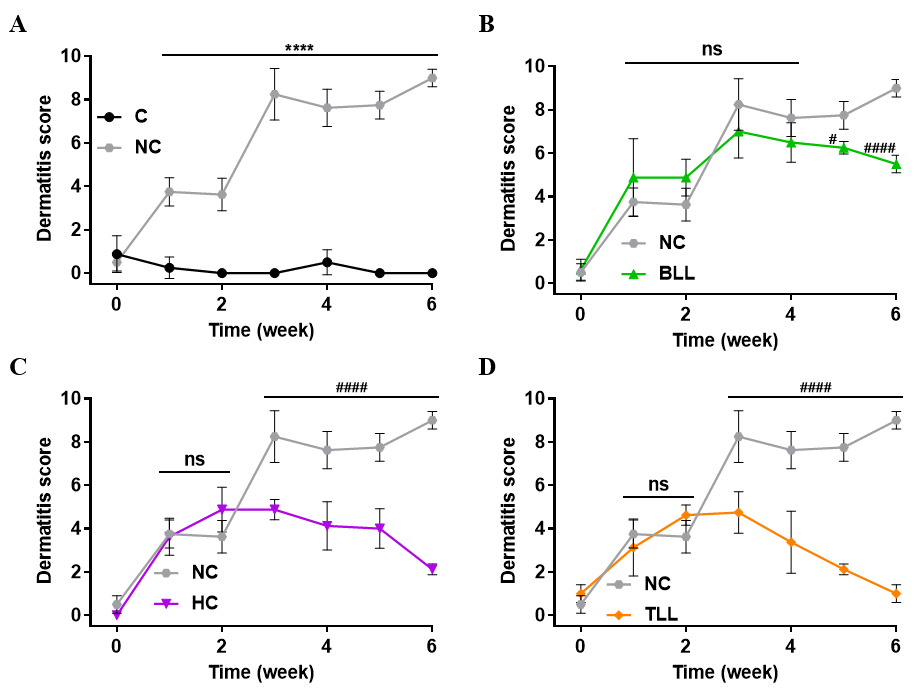


**Fig. S7.** Dermatitis scores in AD-induced mice over 6 weeks. (A) comparison between C and NC groups; (B) comparison between BLL and NC groups; (C) comparison between HC and NC groups; (D) comparison between TLL and NC groups. Values are represented as mean ± SD (n=4). *** *P* < 0.001 vs C, **** *P* < 0.0001 vs C, ^#^ *P* < 0.05 vs NC, ^####^ *P* < 0.0001 vs NC. If it is not statistically significant, it is indicated as ns.

**
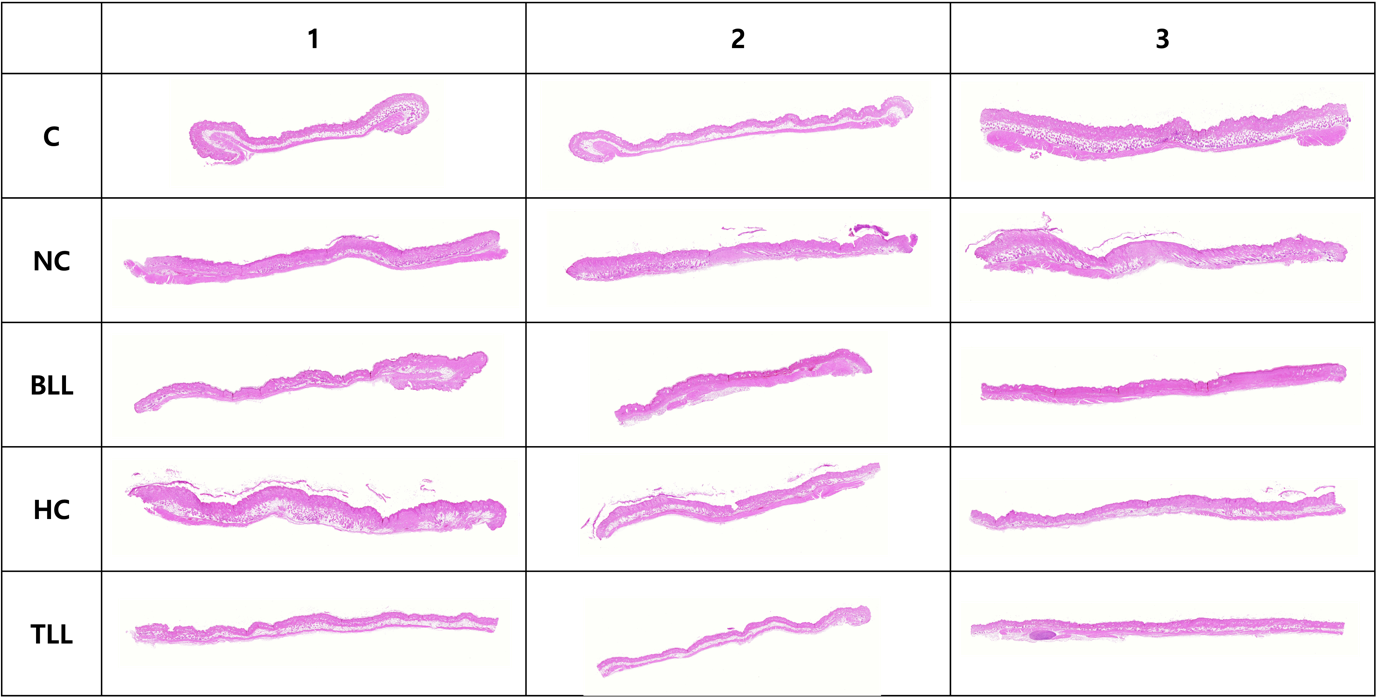
**

**
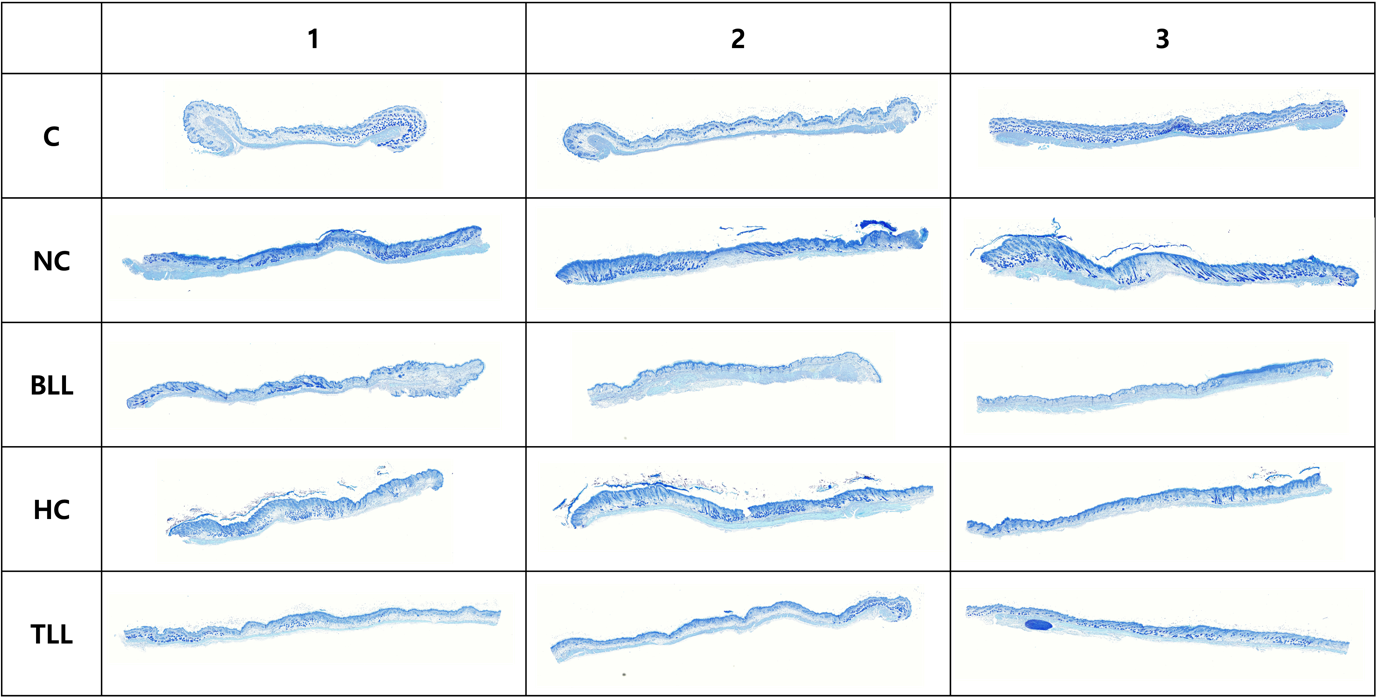
**

**Fig. S8.** Representative H&E- and toluidine blue-stained skin tissue images from individual mice in the AD model. Five groups were included: Control, Negative control (NC), 1.0% hydrocortisone (HC), BLL, and TLL.
